# Flow cytometric evaluation of physico-chemical impact on Gram-positive and Gram-negative bacteria

**DOI:** 10.3389/fmicb.2015.00939

**Published:** 2015-09-24

**Authors:** Antje Fröhling, Oliver Schlüter

**Affiliations:** Quality and Safety of Food and Feed, Leibniz Institute for Agricultural Engineering Potsdam-Bornim e.V.Potsdam, Germany

**Keywords:** culturability, membrane potential, esterase activity, membrane permeabilization, cold atmospheric pressure plasma, peracetic acid, ozonated water

## Abstract

Since heat sensitivity of fruits and vegetables limits the application of thermal inactivation processes, new emerging inactivation technologies have to be established to fulfill the requirements of food safety without affecting the produce quality. The efficiency of inactivation treatments has to be ensured and monitored. Monitoring of inactivation effects is commonly performed using traditional cultivation methods which have the disadvantage of the time span needed to obtain results. The aim of this study was to compare the inactivation effects of peracetic acid (PAA), ozonated water (O_3_), and cold atmospheric pressure plasma (CAPP) on Gram-positive and Gram-negative bacteria using flow cytometric methods. *E. coli* cells were completely depolarized after treatment (15 s) with 0.25% PAA at 10°C, and after treatment (10 s) with 3.8 mg l^−1^ O_3_ at 12°C. The membrane potential of CAPP treated cells remained almost constant at an operating power of 20 W over a time period of 3 min, and subsequently decreased within 30 s of further treatment. Complete membrane permeabilization was observed after 10 s O_3_ treatment, but treatment with PAA and CAPP did not completely permeabilize the cells within 2 and 4 min, respectively. Similar results were obtained for esterase activity. O_3_ inactivates cellular esterase but esterase activity was detected after 4 min CAPP treatment and 2 min PAA treatment. *L. innocua* cells and *P. carotovorum* cells were also permeabilized instantaneously by O_3_ treatment at concentrations of 3.8 ± 1 mg l^−1^. However, higher membrane permeabilization of *L. innocua* and *P. carotovorum* than of *E. coli* was observed at CAPP treatment of 20 W. The degree of bacterial damage due to the inactivation processes is highly dependent on treatment parameters as well as on treated bacteria. Important information regarding the inactivation mechanisms can be obtained by flow cytometric measurements and this enables the definition of critical process parameters.

## Introduction

Perishable products are naturally contaminated with microorganisms including human pathogenic and spoilage bacteria. The level of contamination depends on the season and on the type of fruit and vegetables and ranges between 3 and 7 log units per gram (Ölmez and Kretzschmar, 2009). The microbial load of perishables with human pathogenic bacteria is divers and often leads to foodborne diseases. The microbial load does not only consist of human pathogenic bacteria, there are also phyto pathogenic bacteria present on the fresh produce which can result in postharvest losses or reduced consumer acceptance due to reduced shelf-life. Kader (2005) estimated that one third of all fruits and vegetables produced is not consumed by humans. Throughout the world, the amount of postharvest losses is depending on commodities, production areas, and seasons (Kader, 2005). To minimize the risk of foodborne diseases and to improve microbial quality, decontamination of fresh produce is necessary. Washing of fruits and vegetables removes soil and dirt from the produce but only reduces microbial load by 1 or 2 log units (Sapers, 2001). But washing can also be a potential source for microbial contamination of produce, e.g., pathogens can easily be distributed by the wash water along the processing chain (Doyle and Erickson, 2008). An additional 10- to 100-fold reduction of bacterial load can be achieved using disinfectants, but some disinfectants are only suitable for equipments, others can be used in direct washing of products (Beuchat, 1998). Thermal treatments are limited both by the requirements for the appropriate inactivation of pathogens demanding for temperatures far above 45°C, and the negative impact on produce quality at these elevated temperatures (Schlüter et al., 2009). The disinfection of produce is therefore limited to non-thermal processes below 45°C. Consequently, new emerging inactivation technologies have to be established to fulfill the requirements of food safety, whereas detailed knowledge about these inactivation methods is necessary to ensure efficacy of inactivation processes.

Peracetic acid (PAA) is also known as peroxyacetic acid, acetyl hydroxide or ethaneperoxoic acid. The peroxide of acetic acid is a strong oxidant and disinfectant. The commercially available PAA is a quaternary equilibrium mixture that consists of acetic acid, hydrogen peroxide, PAA, and water (Kitis, 2004). PAA can be used over a wide temperature range (0–40°C), the efficiency of PAA is not affected by protein residues, it can be used with hard water, in cleaning-in-place processes and carbon dioxide saturated environments. Additionally, it is efficient over a wide range of pH (3.0–7.5) (Kunigk and Almeida, 2001). The decomposition products of PAA are water, oxygen, hydrogen peroxide, and acetic acid (Kitis, 2004; Wang et al., 2006). In the food processing industry (e.g., dairies, wineries, breweries, canneries, meat and poultry-processing plants, and beverage industry) peracetic acid (PAA) is used as disinfectant and for wastewater disinfection (Kitis, 2004; Luukkonen et al., 2014). PAA is used as disinfectant and recoloring agent in the pulp, textile, and paper industry (Koivunen and Heinonen-Tanski, 2005; El Shafie et al., 2009; Rasimus et al., 2011) as well as for the disinfection of ion exchangers and cooling towers and for pathogen reduction in sludge debulking, biosolids, and for the reduction of solid odors (Kitis, 2004; Koivunen and Heinonen-Tanski, 2005). The efficiency of PAA against bacteria on fresh fruits and vegetables as well as on fresh-cut fruits and vegetables was examined in various studies (Beuchat et al., 2004; Kim et al., 2006; Alvaro et al., 2009; Vandekinderen et al., 2009; Van De Velde et al., 2014).

With an oxidizing potential of 2.07 V, ozone is the fifth in thermodynamic oxidation potential behind fluorine, chlorine trifluoride, atomic oxygen, and hydroxyl free radical (Graham, 1997). This oxidizing potential makes ozone the strongest disinfection agent available for the contact with foods (Mahapatra et al., 2005) and water and wastewater treatment (Graham, 1997). The solubility of ozone in water is dependent on several parameters. The temperature of the water, the bubble size and the presence of minerals or organic matter highly influences the solubility of ozone. The half-life of ozone in gaseous state is 12 h at room temperature and in pure, clean water (pH 7–8) the half-life is 20–30 min (Khadre and Yousef, 2001; Kim et al., 2003). The decomposition of ozone in solution is a stepwise fashion accompanied by the production of free radicals such as hydroperoxyl, hydroxyl, and superoxide. These free radicals have a great oxidizing power and the reactivity of ozone is attributed to these radicals. In the food industry ozone applications are related to decontamination of product surfaces, food plant equipment, reuse of waste water, and lowering biological oxygen demand (BOD) and chemical oxygen demand (COD) of food plant waste. Inactivation of contaminated microflora on meat, poultry, eggs, fish, fruits, vegetables, and dry food by ozone was met with mixed success (Guzel-Seydim et al., 2004). In fruit and vegetable processing ozone is applied in gaseous state and dissolved in water to improve food safety (Tiwari, 2012). Overviews of studies focusing on decontamination of food products by ozone are given by Perry and Yousef (2011), O'Donnell et al. (2012), and Miller et al. (2013).

Plasma treatment in which the treated surface temperature is kept below the temperature of thermal treatments can be defined as cold plasmas (Schlüter and Fröhling, 2014). Atmospheric-pressure plasma sources used for microbial decontamination are commonly generated by corona discharge, dielectric barrier discharge, atmospheric pressure plasma jet, and MW-driven plasmas (Ehlbeck et al., 2011). Cold plasma treatment is of grown interest in food microbiology and in various studies the antimicrobial activity of cold plasma against Gram-negative and Gram-positive bacteria, yeast and fungi, biofilm formers, and endospores as well as the effects of plasma on biomolecules such as proteins and enzymes was shown (Laroussi, 2005; Vleugels et al., 2005; Brandenburg et al., 2007; Surowsky et al., 2013; Bußler et al., 2015a,b; Misra et al., 2015). The inactivation of food-related microorganisms by plasma treatment is mainly performed using model systems but the evaluation of plasma effects on microorganisms attached to food surfaces is more and more of interest. Surowsky et al. (2014b) summarized the interactions of plasma detected on liquid and solid food surfaces. Although several reviews focus on the inactivation mechanisms of plasma (Moisan et al., 2001; Boudam et al., 2006; Gaunt et al., 2006; Moreau et al., 2008), the inactivation mechanism and its potential regarding mild food surface decontamination is up to now not fully understood because the inactivation effects are depending on the applied plasma sources and experimental conditions. The presence of free radicals, UV emitting species, and charged particles is associated with the antimicrobial effect of the plasma (Laroussi, 2002; Moisan et al., 2002). Proposed inactivation effects are summarized by Schlüter and Fröhling (2014).

Detailed knowledge of inactivation effects is required for successful implementation of inactivation treatments in the production chain. Furthermore, the success of the inactivation process has to be verified online to ensure product safety. Due to the short shelf-life of fresh produce the results need to be obtained within a limited time period to enable contamination-related process control. Conventional microbiological techniques such as plate count methods are very time consuming and an absence of culturability cannot be directly related to cellular death because the bacteria can still be metabolically active (Bunthof and Abee, 2002). Therefore, the implementation of these methods in the processing chain is limited. In response to these limitations minimal duration methods to monitor inactivation treatments are required. Flow cytometry is a promising tool in food microbiology as it enables measurements on a single cell level and the detection of physiological property changes of bacteria after certain treatment processes within a short time due to the development of appropriate fluorescent dyes and improvement of optic technology. Fluorescent dye technology offers probes for a variety of cellular functions (Joux and Lebaron, 2000; Johnson et al., 2013). The use of fluorescent dye mixtures enables the classification into three types of viable cells: metabolically active, intact, or permeabilized cells (Hewitt and Nebe-Von-Caron, 2004). The types of fluorescent dyes primary used in flow cytometry are: (i) fluorescent immune-conjugates and probes for fluorescence *in situ* hybridization; (ii) nucleic acid strains; and (iii) physiological probes to measure ions, membrane potential, enzymatic activity, viability, organelles, phagocytosis, cell development, and other cell properties (Haugland, 1994). The potential of flow cytometry to assess yeast cultures in food and beverage processing (e.g., bakery, wine industry, beer industry) is already shown (Herrero et al., 2006) as well as used to monitor the solid-state fermentation of basidiomycetes (Steudler et al., 2015). Flow cytometry is used in various studies to monitor bacterial inactivation (Luscher et al., 2004; Ananta et al., 2005; Berney et al., 2007; Mathys et al., 2007; Ananta and Knorr, 2009; Da Silveira and Abee, 2009; Joyce et al., 2011; Schenk et al., 2011; Fröhling et al., 2012b; Tamburini et al., 2013; Bigoni et al., 2014).

In this study, flow cytometry is used to investigate inactivation effects of peracetic acid, ozonated water, and cold atmospheric pressure plasma and to prove the ability of flow cytometric techniques for short-time monitoring of inactivation processes. For this purpose morphological and physiological properties of bacteria (membrane integrity and RNA/DNA damage, esterase activity, and membrane potential) were determined by flow cytometry. Various bacteria associated with food contamination are selected in order to examine the inactivation effects on different types of microorganisms. *Listeria innocua* (Gram-positive) is chosen as indicator strain for the human pathogenic *Listeria monocytogenes* (Kamat and Nair, 1996), a non-pathogenic *Escherichia coli* (Gram-negative) strain is chosen as representative for fecal contamination, and the plant pathogenic strain *Pectobacterium carotovorum* (Gram-negative) is chosen as a spoilage bacterium.

## Material and methods

### Cultivation of bacteria

*Listeria innocua* (DSM 20649), *Escherichia coli* (DSM 1116), and *Pectobacterium carotovorum* spp. *carotovorum* (DSM 30168) were stored as glass bead cultures at −80°C for long-term maintenance. One glass bead was given to 5 ml nutrient broth (Carl Roth GmbH & Co KG, Germany) and incubated for 24 h without shaking at 37°C (*L. innocua, E. coli*) or 30°C (*P. carotovorum*) to re-activate the bacteria. Afterwards, 100 ml nutrient broth was inoculated with bacteria suspension at a calculated optical density at 620 nm (OD_620_) of 0.07 ml^−1^. Bacteria were harvested in the early to mid-stationary growth phase (*L. innocua* and *E. coli:* 18 h at 37°C or *P. carotovorum*: 18 h at 30°C) by centrifugation at 3220 × *g* for 15 min at 4°C. The pelleted material was suspended in 50 mM phosphate buffered saline (PBS) to a cell concentration of approximately 9–10 log CFU ml^−1^. PBS was prepared of 137 mM NaCl, 2.7 mM KCl, 40.6 mM Na_2_HPO_4_, and 7.1 mM KH_2_PO_4_. The pH was adjusted to 7.0 with HCl and finally filtered with a 0.2 μm membrane filter. All reagents were provided by Carl Roth GmbH & Co KG, Germany.

### Treatment with peracetic acid

Wofasteril® E400 (Kesla, Germany) was used as peracetic acid solution (PAA). Immediately before the treatments PAA concentration of 0.25% was prepared using tap water and tempered to 10°C in a water bath. The treatment volume was 10 ml temperate PAA solution with an initial bacterial count of approximately 7 log CFU ml^−1^. The treatment times were 0.25, 0.5, 0.75, 1, 1.5, and 2 min. To stop the reaction after the defined treatment times the solution was added to 30 ml 50 mM PBS containing 0.6 M sodium thiosulfate (Na_2_S_2_O_3_) and mixed for 10 s. As a control bacteria were treated with tempered tab water. After treatment the samples were centrifuged at 3220 × *g* for 15 min at 4°C and afterwards the pelleted material was re-suspended in 10 ml 50 mM PBS for analyses of inactivation effects. It was also tested, whether Na_2_S_2_O_3_ also lead to an inactivation of bacteria. For that, 10 ml bacteria solution was added to 30 ml PBS buffer containing 0.6 M Na_2_S_2_O_3_. Effects of Na_2_S_2_O_3_ on bacteria were detected by plate count method and were not significant. Each treatment was performed in triplicate.

### Treatment with ozonated water

A Bewazon 1 ozone generator (BWT Wassertechnik GmbH, Germany) was used to generate ozonated water. The ozone concentration in mg l^−1^ was measured in the spectrophotometer DR2800 (Hach Lange, Germany) using the LCK310 Chlorine/Ozone cuvette test (Hach Lange, Germany) following the instructions of the producer. Ozonated water with an ozone concentration of 3.8 ± 1.4 mg l^−1^ was used for inactivation treatments. The treatment volume was 10 ml ozonated water with an initial bacterial count of approximately 7 log CFU ml^−1^. The treatment time was set to 0.17, 0.5, 1, 1.5, and 2 min. To stop the reaction the sample tubes were intensively shaken for approximately 30 s [O_3_(–)]. To prove an inappropriate removal of residual ozone and to avoid false positive inactivation of bacteria, Na_2_S_2_O_3_ was used to stop the reaction in a second trial. Therefore, the bacteria were treated with ozonated water using an ozone concentration of 3.8 ± 1.02 mg l^−1^ as described above and the reaction was stopped with Na_2_S_2_O_3_ [O_3_(+)]. After the defined treatment time 44.1 mM Na_2_S_2_O_3_ was added to the sample tubes and the tubes were shaken for 2–3 s to allow a homogenous distribution of Na_2_S_2_O_3_ within the sample. An inactivation effect of Na_2_S_2_O_3_ was excluded in preliminary experiments. After treatment the samples were centrifuged at 3220 × *g* for 15 min at 4°C and afterwards the pelleted material was re-suspended in 50 mM PBS for analyses of inactivation effects. Each treatment was performed in triplicate.

### Treatment with cold atmospheric pressure plasma

The used plasma source and sample preparation is described in detail by Fröhling et al. (2012a). The distribution of reactive species was previously determined for a similar CAPP jet using Optical Emission Spectroscopy (Brandenburg et al., 2007). The initial bacterial concentration of each gel disc was approximately 8 log CFU cm^−2^. For plasma treatment the gel samples were placed in the center of a glass sample holder and treated between 0.25 and 4 min. The operating power of the plasma jet was set to 20 W. The distance between the nozzle and the sample was 10 mm so that the plasma covered the inoculated region completely. The temperature of the samples during plasma treatment was recorded using a thermography camera (SC500, Flir Systems GmbH, Germany). As control inoculated gel sample were handled as described but without plasma treatment. Each treatment was performed in triplicate. Treated and untreated gel samples were transferred into sample tubes, and stored over night at 4°C before analysis due to logistic reasons. Effects of overnight storage on the obtained results were excluded in preliminary tests. To re-suspend the bacteria, 1 ml PBS (50 mM, pH 7) were added to each sample tube and agitated for 5 min at 750 rpm and 37°C. The obtained bacteria suspension was transferred to sample tubes for plate count analysis and flow cytometric sample preparation.

### Flow cytometric analysis

All experiments were performed using a Cytomics FC 500 flow cytometer (Beckman Coulter, Germany) equipped with a 20 mW argon ion laser emitting at a wavelength of 488 nm. The fluorescence of thiazole orange, carboxyfluorescein, and green DiOC_2_(3) was collected in the FL1 photomultiplier with a band pass filter of 525 ± 25 nm and the fluorescence of propidium iodide and red DiOC_2_(3) was recorded in the FL3 photomultiplier with a short pass filter of 620 nm. Fluorescence compensation was performed to correct the overlap of one dye's emission into another dye's detector. The parameters were collected as logarithmic signals and the obtained data were analyzed using CXP Analysis software (Beckman Coulter, Germany). Ten thousand events were measured at a flow rate of approximately 300 events s^−1^. Unless otherwise stated, the density plots obtained by flow cytometric analyses were divided into four regions. The regions represent cells with different physiological properties. The average of the percentage values obtained from three density plots was calculated and illustrated as kinetics in diagrams where the x-coordinate displays the treatment time and the y-coordinate the percentage of stained cells.

#### Membrane potential

3,3′-diethyloxacarbocyanine iodide [DiOC_2_(3)] was applied to measure the membrane potential of bacteria cells. The staining was performed according to Novo et al. (1999) with small modifications. DiOC_2_(3) was provided by Sigma-Aldrich, Germany. For staining with DiOC_2_(3) the bacteria suspension was diluted in 50 mM PBS containing 20 mM D-glucose to achieve a cell concentration of approximately 6 log cells ml^−1^. Then, 30 μM DiOC_2_(3) was added and incubated for 15 min in the dark at room temperature. Afterwards the suspension was centrifuged at 4000 × *g* and 4°C for 6 min (Gram-negative bacteria) or 12,000 × *g* for 10 min at 4°C (Gram-positive bacteria). The pelleted material was re-suspended in 50 mM PBS to a cell density of approximately 6 log cells per ml and immediately measured in the flow cytometer.

Cells were completely depolarized with carbonyl cyanide m-chlorophenylhydrazone to set the parameters of the flow cytometer. The settings of the FL1 [green DiOC_2_(3)-fluorescence intensity] and FL3 [red DiOC_2_(3)-fluorescence intensity] photomultiplier were chosen so that the mean fluorescence of green and red DiOC2(3) was detected in the same channel. The ratio of mean red to mean green DiOC2(3)- fluorescence channel value was calculated to investigate changes in the membrane potential. Due to the chosen cytometer settings the red/green DiOC2(3)-fluorescence ratio of depolarized cells was ≤ 1. It was assumed that the red/green ratio of untreated cells represents the relative membrane potential of intact cells (Novo et al., 1999). A reduction of the red/green ratio stands for the loss cell membrane potential.

#### Membrane integrity and RNA/DNA damage

Thiazole orange (TO) and Propidium iodide (PI) were used to distinguish between RNA and DNA in bacteria cells as well as to indicate compromised cell membranes and DNA damage. Therefore, 0.42 μM TO and 30 μM PI were added to a bacteria suspension containing ~6 log cells ml^−1^ and incubated for 10 min at room temperature in the dark before flow cytometric measurements. Due to the fact that the TO-RNA-complex shows lower fluorescence intensities than the TO-DNA-complex (Nygren et al., 1998) the mean value of TO-fluorescence intensity was used to distinguish between RNA and DNA staining.

#### Esterase activity and membrane permeabilization

The bacteria suspension was centrifuged at 4000 × *g* and 4°C for 6 min (Gram-negative bacteria) or 12,000 × *g* for 10 min at 4°C (Gram-positive bacteria) after inactivation treatment. The pelleted material was re-suspended in PBS (50 mM, pH 7) to obtain a calculated OD_620_ of approximately 10 for staining procedure. Fifty micromole cFDA (Gram-positive bacteria) or 0.83 mM cFDA (Gram-negative bacteria) was added to the bacterial suspension in the sample tubes and allowed to penetrate into the cells for 15 min (Gram-positive bacteria) or 45 min (Gram-negative bacteria) in a water bath set to 37°C. Afterwards, surplus cFDA was removed by centrifugation for 6 min at 4000 × *g* and 4°C or 12,000 × *g* for 10 min at 4°C. The pelleted material was re-suspended in 50 mM PBS to obtain a cell concentration of approximately 6 log cells ml^−1^. To investigate membrane integrity of bacteria cells 30 μM propidium iodide was added to cFDA treated cells and allowed to penetrate into permeabilized cells for 10 min at 4°C in the dark before flow cytometric measurements.

#### Total viable cell count

The viable cell count of bacteria after inactivation treatments was determined by traditional culture methods in duplicate. Therefore, the samples were serially diluted in Rotilabo®-microtest plates (96er U-profile, Carl Roth GmbH & Co KG, Germany) using 50 mM PBS as dilution solution. 100 μl of each dilution was spread on the cultivation medium. The number of colony forming units (CFU) was evaluated after 48 h at 37°C (*L. innocua*, Standard-I agar: Carl Roth GmbH & Co KG, Germany); 48 h at 30°C (*P. carotovorum*, MacConkey agar: Carl Roth GmbH & Co KG, Germany); or 24 h at 37°C (*E. coli*, Standard-I agar: Carl Roth GmbH & Co KG, Germany). The detection limit of plate count analyses was 1 log CFU ml^−1^ for treatment with peracetic acid and ozonated water and 1 log CFU cm^−2^ for treatment with cold atmospheric pressure plasma.

### Mathematical modeling and statistical analysis

To obtain an inactivation kinetic for each treatment parameter, the average of six values of colony forming units were calculated (three independent experiments analyzed in duplicate). The inactivation kinetics obtained were modeled with the log-linear regression model with tailing using GInaFiT (Geeraerd and Van Impe Inactivation Model Fitting Tool), a freeware Add-in for Microsoft® Excel (Geeraerd et al., 2005).

The GInaFiT tool was also applied to the obtained flow cytometric data. The relative membrane potential was modeled with the log-linear regression model with tailing; esterase activity and the decrease of intact cells were described by the log-linear regression. Additionally, membrane permeabilization kinetics were modeled with a Gompertz model using the software SigmaPlot13 (Systat Software Inc.).

Statistical analyses to evaluate significant difference between the data were performed using Origin® 7.5 software. One Way-ANOVA with Tukey test and a significance level of 0.05 were used.

## Results

### Treatment effects on total viable count

For all tested bacteria the peracetic acid treatment was applied at 10°C and the temperature of the ozonated water was approximately 12°C. Temperature measurements at 20 W CAPP showed that the average temperature of the gel discs slightly increased to approximately 27°C (data not shown).

**Figure 1:**
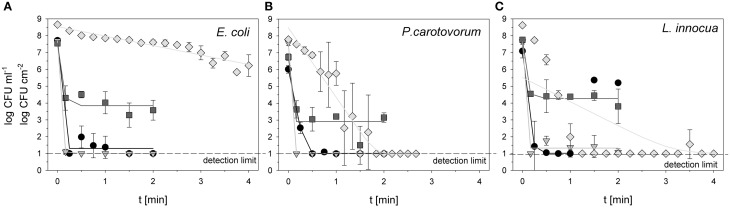
**Total viable count of ***E. coli*** (A), ***P. carotovorum*** (B), and ***L. innocua*** (C) after PAA treatment (circles: 10°C, 0.25% PAA), CAPP treatment (rhombus: 20 W), and treatment with ozonated water [triangle: 3.8 ± 1.02 mg l^−1^ O_3_(–); square: 3.8 ± 1.02 mg l^−1^ O_3_(+)]**. The lines represent the inactivation obtained by the GInaFit tool.

The initial count of *E. coli* for PAA treatment was 7.72 log CFU ml^−1^. The treatment with 0.25% PAA decreased the viable count to the detection limit of 1 log CFU ml^−1^ within 0.25 min treatment time. With increasing treatment time to 0.5, 0.75, and 1 min the total viable count was between 1.37 and 1.98 CFU ml^−1^ and decreased again to the detection limit after 1.5 and 2 min treatment time (Figure 1A). For treatment with ozonated water the initial count of *E. coli* was 7.71 and 7.54 log CFU ml^−1^. The treatment with an ozone concentration of 3.8 mg l^−1^ O_3_(–) led to a reduction of bacteria to the detection limit of 1 log CFU ml^−1^ after a treatment time of 0.17 min (Figure 1A). Using an ozone concentration of 3.78 mg l^−1^ O_3_(+) led to a decrease of total viable count to 3.58 log CFU ml^−1^ within 2 min (Figure 1A). For CAPP treatment the initial load of *E. coli* was 8.66 log CFU cm^−2^. Within 4 min the total viable count of *E. coli* was reduced to 6.22 log CFU cm^−2^ (Figure 1A).

6.02 log CFU ml^−1^ was used as initial count of *P. carotovorum* for the treatment with 0.25% PAA. The total viable count of *P. carotovorum* was decreased to the detection limit of 1 log CFU ml^−1^ within 0.5 min treatment time (Figure 1B). Using ozonated water at a concentration of 2.8 mg l^−1^ O_3_(–), the total viable count of *P. carotovorum* was reduced from 7.6 log CFU ml^−1^ to the detection limit of 1 log CFU ml^−1^ within 0.17 min (Figure 1B). Within 2 min the total viable count of *P. carotovorum* was only reduced from 6.74 to 3.15 log CFU ml^−1^ using an ozone concentration of 3.82 mg l^−1^ O_3_(+) (Figure 1B). The treatment with CAPP reduced the total viable count of *P. carotovorum* from 7.79 log CFU cm^−2^ to the detection limit of 1 log CFU cm^−2^ within 1.5 min (Figure 1B).

The initial count of *L. innocua* for treatment with 0.25% PAA was 7.09 log CFU ml^−1^. Within 0.75 min the total viable count of *L. innocua* was reduced to the detection limit of 1 log CFU ml^−1^ but increased again to 5.36 and 5.20 log CFU ml^−1^ after 1.5 and 2 min, respectively (Figure 1C). The initial count of 7.65 CFU ml^−1^ was reduced to 1.08 log CFU ml^−1^ with 2 min treatment with ozonated water and an ozone concentration of 3.42 mg l^−1^ O_3_(–). The total viable count of *L. innocua* was only reduced from 7.79 to 3.81 log CFU ml^−1^ within 2 min using 3.8 mg l^−1^ O_3_(+) (Figure 1C). The treatment with CAPP led to a microbial reduction from 8.63 log CFU cm^−2^ to the detection limit of 1 log CFU cm^−2^ within 1.25 min (Figure 1C).

### Effects of treatments on membrane potential

**Figure 2:**
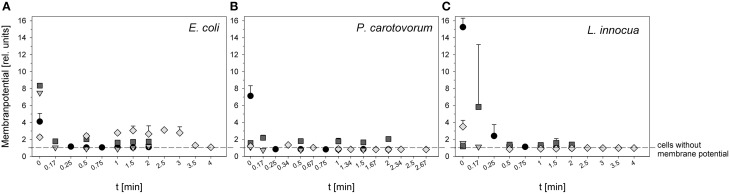
**Relative membrane potential of ***E. coli*** (A), ***P. carotovorum*** (B), and ***L. innocua*** (C) after PAA treatment (circles: 10°C, 0.25% PAA), CAPP treatment (rhombus: 20 W), and treatment with ozonated water [triangle: 3.8 ± 1.02 mg l^−1^ O_3_(–); square: 3.8 ± 1.02 mg l^−1^ O_3_(+)] expressed as red/green ratio of DiOC_2_(3)-fluorescence intensity**.

The measurement of relative membrane potential using DiOC_2_(3) showed that the untreated *E. coli* cells had a red/green ratio of 8.8 before treatment with 0.25% PAA. After PAA treatment the red/green ratio was reduced to approximately 1.0 regardless of the treatment time indicating a reduced membrane potential (Figure 2A). The relative membrane potential of *E. coli* was also immediately reduced from 7.5 to approximately 1 using ozonated water with an ozone concentration of 3.8 mg l^−1^ O_3_(–) (Figure 2A). However, using the same ozone concentration but stopping the reaction with Na_2_S_2_O_3_ [O_3_(+)] only decreased the relative membrane potential from 8.4 to approximately 2 indicating a higher relative membrane potential of *E. coli* cells than after PAA treatment and ozone treatment where the reaction is stopped by shaking (Figure 2A). The initial value of the relative membrane potential of *E. coli* cells was approximately 2 before plasma treatment and remained almost constant after treatment with CAPP regardless of the treatment time. Only after 3 min treatment time a reduction of membrane potential could be detected (Figure 2A).

The cells of *P. carotovorum* were immediately depolarized from 7.13 to < 1 after treatment with 0.25% PAA regardless of treatment time (Figure 2B). *P. carotovorum* cells after treatment were also depolarized after treatment with 2.8 mg l^−1^ O_3_(–). However, the initial relative membrane potential was only 1.08 and was reduced to < 1 after 0.17 min (Figure 2B). A slightly higher relative membrane potential (1.58) was detected before treatment with 3.82 mg l^−1^ O_3_(+) but the relative membrane potential remained almost constant during 2 min treatment (Figure 2B). The treatment with CAPP reduced the relative membrane potential from 1.2 to < 1 within 1 min treatment (Figure 2B).

*L. innocua* cells had a relative membrane potential of 15.25 before treatment with 0.25% PAA. The cells were completely depolarized after 1 min treatment (Figure 2C). Using ozonated water with an ozone concentration of 3.42 mg l^−1^ O_3_(-) reduced the relative membrane potential from 1.57 to < 1 within 1.5 min (Figure 2C). In contrast, 3.8 mg l^−1^ O_3_(+) increased the relative membrane potential from 1.19 to 5.82 within 0.17 min and with increasing treatment time the relative membrane potential decreased again to approximately 1.5 and remained almost constant during treatment up to 2 min (Figure 2C). The relative membrane potential of *L. innocua* cells was immediately reduced from 3.51 to < 1 within 1 min treatment with CAPP (Figure 2C).

### Treatment effects on membrane integrity and bacterial RNA and DNA

**Figure 3:**
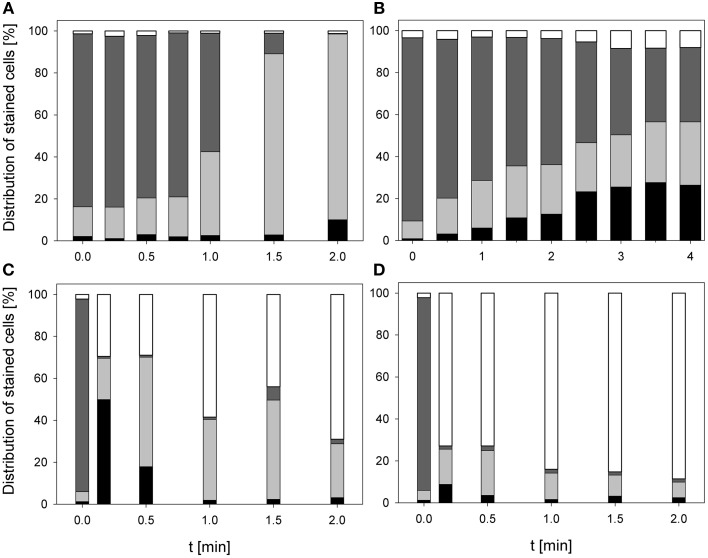
**Membrane integrity of ***E. coli*** after PAA treatment [(A) 10°C, 0.25% PAA], CAPP treatment [(B) 20 W), and treatment with ozonated water [(C) 3.8 mg l^−1^ O_3_(–); (D) 3.78 mg l^−1^ O_3_(+)]**. Black bars representing permeabilized cells; gray bars representing slightly permeabilized cells, light gray bar representing intact cells, and white bars representing cells without fluorescence/cell fragments.

During treatment with 0.25% PAA the percentage of permeabilized *E. coli* cells increased to 10% within 2 min and the percentage of slightly permeabilized cells increased to 88.6%. The percentage of intact cells decreased to 0% within 2 min and the population of unstained cells/cell fragments remained below 3% within the treatment time (Figure 3A). During 0.25% PAA treatment the mean TO-fluorescence intensity significantly increased within the first 0.5 min and then significantly decreased again with increasing treatment time (Table 1). The treatment with CAPP led to an increase of permeabilized *E. coli* cells to approximately 27% within 4 min. The percentage of slightly permeabilized cells also continuously increased to 30% within 4 min whereas the percentage of intact cells simultaneously decreased to 35%. The population of unstained cells/cell fragments remained almost constant at approximately 3–4% within the first 2 treatment minutes. A further increase of treatment time led to an increase of unstained cells/cell fragments to approximately 8% (Figure 3B). During treatment with CAPP the mean TO-fluorescence intensity significantly increased in the first 0.5 min and then remained almost constant with increasing treatment time (Table 1). 3.78 mg l^−1^ O_3_(–) led to an increase of permeabilized *E. coli* cells to 49.8% within 0.17 min and with increasing treatment time up to 2 min the percentage of permeabilized cells decreased again to 3.1%. Concurrently, the percentage of intact cells decreased immediately to < 1% within 0.17 min and remained almost constant at this level. However, after 1.5 min ozone treatment the percentage of intact cells increased again to 6.3% and decreased to 2.2% after 2 min. The percentage of slightly permeabilized cells increased within 0.5 min of treatment to 52.3% and decreased again with increasing treatment time to 25.6% (Figure 3C). The treatment with 3.8 mg l^−1^ O_3_(+) only slightly increased the percentage of permeabilized *E. coli* cells to 8.3% within 0.17 min and with increasing treatment time the percentage decreased again to 2.5%. The number of slightly permeabilized cells increased to 21.4% within 0.5 min and decreased again to 7.4% with increasing treatment time. The applied ozone concentration led to an immediate decrease of intact cells to < 2% within 0.17 min whereas the population of unstained cells/cell fragments immediately increased to 72.9% within 0.17 min and continuously increased to 88.6% applying treatment times up to 2 min (Figure 3D). *E. coli* showed a significant decrease of mean TO-fluorescence intensity after both applied ozone treatments (Table 1).

**Table 1:** **Mean TO-fluorescence intensity of ***E. coli*** after different treatments**.

**t [min]**	**0.25% PAA**			**3.8 mg l**^**−1**^ **O**_**3**_ **(-)**			**3.78 mg l^−1^ O_3_ (+)**			**20 W CAPP**		
	**Mean TO-fluorescence intensity [mV]**		***SD***	**Mean TO-fluorescence intensity [mV]**		***SD***	**Mean TO-fluorescence intensity [mV]**		***SD***	**Mean TO-fluorescence intensity [mV]**		***SD***
0	823.9^a^^,^^d^^,^^e^		± 108.1	1455.7^a^		± 35.2	328.9^a^		± 24.6	254.0^a^		± 29.3
0.17		n.d.		149.8^b^		± 58.9	84.7^b^		± 11.3		n.d.	
0.25	523.7^b^		± 33.4		n.d.			n.d.			n.d.	
0.5	1094.2^a^^,^^c^^,^^d^		± 27.6	84.7^b^		± 14.9	97.7^b^		± 33.8	573.2^b^		± 14.9
0.75	1169.1^c^^,^^d^^,^^e^		± 114.1		n.d.			n.d.			n.d.	
1	1003.1^d^^,^^e^		± 20.3	94.4^b^		± 11.3	94.4^b^		± 5.6	540.6^b^		± 66.5
1.5	768.6^e^		± 148.3	127.0^b^		±16.9	91.2^b^		± 14.9	501.5^b^		± 20.3
2	312.6^b^		± 42.6	107.5^b^		± 19.5	136.8^b^		±16.9	491.8^b^		± 14.9
2.5		n.d.			n.d.			n.d.		452.7^b^		± 29.8
3		n.d.			n.d.			n.d.		436.4^b^		± 73.3
3.5		n.d.			n.d.			n.d.		511.3^b^		±53.8
4		n.d.			n.d.			n.d.		527.6^b^		± 112.7

*SD, Standard deviation of three independent experiments*.

a−e *Different letters within the lines indicate significant differences at a significance level of 0.05*.

**Figure 4:**
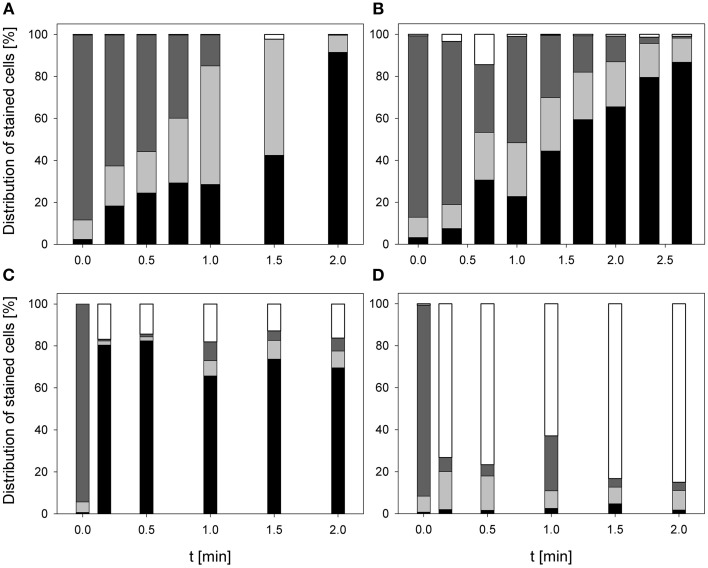
**Membrane integrity of ***P. carotovorum*** after PAA treatment [(A) 10°C, 0.25% PAA], CAPP treatment [(B) 20 W], and treatment with ozonated water [(C) 2.8 mg l^−1^ O_3_(–); (D) 3.8 mg l^−1^ O_3_(+)]**. Black bars representing permeabilized cells; gray bars representing slightly permeabilized cells, light gray bar representing intact cells, and white bars representing cells without fluorescence/cell fragments.

The treatment with 0.25% PAA led to a continuous increase of permeabilized *P. carotovorum* cells to 91.5% within 2 min of treatment. The percentage of slightly permeabilized cells increased to 56.5% within 1 min of treatment and decreased again to 8.1% after 1.5 min. The number of intact cells decreased during 2 min treatment with PAA to 0.1% and the population of unstained cells/cell fragments remained almost constant < 2% during the PAA treatment time of 2 min (Figure 4A). During 0.25% PAA treatment the mean TO-fluorescence intensity significantly increased in the first 0.25 min and then significantly decreased again with increasing treatment time (Table 2). The percentage of permeabilized *P. carotovorum* cells continuously increased during 2.67 min treatment with CAPP to 86.7% whereas the percentage of intact cells simultaneously decreased to 0.9%. Within the first 0.67 min of treatment, the percentage of unstained cells/cell fragments increased to 14.5% and decreased again with increasing treatment time to 0.9%. The population of slightly permeabilized cells increased to 25.5% within the first 1.34 min and decreased again with increasing treatment time (Figure 4B). The mean TO-fluorescence intensity of *P. carotovorum* did not significantly increase within the first treatment minute but with increasing treatment time to 2 min the mean TO-fluorescence intensity significantly increased. A further increase of treatment time led to a significant reduction of mean TO-fluorescence intensity (Table 2). At 2.8 mg l^−1^ O_3_(–) the percentage of permeabilized *P. carotovorum* cells increased to 82.4% within 0.5 min and decreased again with increasing treatment time to 69.6%. The number of slightly permeabilized cells decreased within the first 0.5 min to approximately 2% but increased again to 8–9% after 2 min. The percentage of intact cells was immediately reduced to < 1% within 0.17 min but increased again to 4–9% within the next 1.5 min of treatment. The population of unstained cells/cell fragments increased to approximately 17% within 0.17 min and remained almost constant at this level with increasing treatment time to 2 min (Figure 4C). The mean TO-fluorescence intensity did not significantly increase during treatment with 2.8 mg l^−1^ O_3_(–) (Table 2). Using 3.82 mg l^−1^ O_3_(+) led only to a minor increase of permeabilized *P. carotovorum* cells. The percentage of intact cells decreased within 0.5 min from 91 to 5.4%, increased again to 26.1% after 1 min and decreased again to 3.9% after 2 min. The population of unstained cells/cell fragments continuously increased during treatment with 3.82 mg l^−1^ O_3_(+) to 85.1% with the exception of 1 min where a decrease to 62.9% was observed. The percentage of slightly permeabilized *P. carotovorum* cells increased up to 18.1% within 0.17 min but decreased again with increasing treatment time to approximately 9% (Figure 4D). Mean TO-fluorescence intensity of *P. carotovorum* showed no significant changes after ozone treatment (Table 2).

**Table 2:** **Mean TO-fluorescence intensity of ***P. carotovorum*** after different treatments**.

**t [min]**	**0.25% PAA**			**2.8 mg l^−1^ O_3_(-)**			**3.8 mg l^−1^ O_3_(+)**			**20 W CAPP**		
	**Mean TO-fluorescence intensity [mV]**		***SD***	**Mean TO-fluorescence intensity [mV]**		***SD***	**Mean TO-fluorescence intensity [mV]**		***SD***	**Mean TO-fluorescence intensity [mV]**		***SD***
0.00	623.5^a^		± 130.3	840.2^a^		± 83.5	1175.7^a^		± 455.8	280.1^a^^,^^c^		± 31.4
0.17		n.d.		706.7^a^		± 225.6	107.5^a^		± 16.9		n.d.	
0.25	997.7^b^		± 90.9		n.d.			n.d.			n.d.	
0.34		n.d.			n.d.			n.d.		351.7^a^^,^^c^		± 48.9
0.50	799.5^b^^,^^c^		± 37.2	696.9^a^		± 395.3	94.4^a^		± 5.6		n.d.	
0.67		n.d.			n.d.			n.d.		602.5^a^^,^^b^^,^^c^		± 297.8
0.75	711.3^a^^,^^c^		± 97.6		n.d.			n.d.			n.d.	
1.00	660.5^a^		± 48.5	1149.6^a^		± 55.6	1234.3^a^		± 1977.1	625.3^a^^,^^b^^,^^c^		± 42.5
1.34		n.d.			n.d.			n.d.		990.0^b^		± 58.9
1.50	172.9^d^		± 62.6	924.9^a^		± 390.4	114.0^a^		± 20.3		n.d.	
1.67		n.d.			n.d.			n.d.		856.5^b^^,^^c^		± 150.2
2.00	22.4^d^		± 0.9	1032.4^a^		± 305.4	110.7^a^		± 11.3	872.8^b^^,^^c^		± 122.2
2.34		n.d.			n.d.			n.d.		648.1^a^^,^^b^^,^^c^		± 198.4
2.67		n.d.			n.d.			n.d.		508.0^c^		± 122.4

*SD, Standard deviation of three independent experiments*.

a–d *Different letters within the lines indicate significant differences at a significance level of 0.05*.

**Figure 5:**
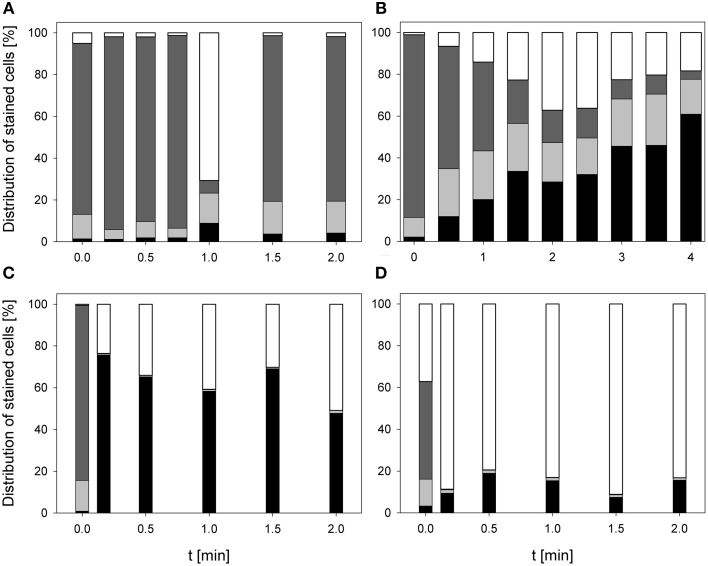
**Membrane integrity of ***L. innocua*** after PAA treatment [(A) 10°C, 0.25% PAA], CAPP treatment [(B) 20 W], and treatment with ozonated water [(C) 3.42 mg l^−1^ O_3_(–); (D) 3.8 mg l^−1^ O_3_(+)]**. Black bars representing permeabilized cells; gray bars representing slightly permeabilized cells, light gray bar representing intact cells, and white bars representing cells without fluorescence/cell fragments.

The treatment with 0.25% PAA did only slightly increase the percentage of permeabilized *L. innocua* cells within the first treatment minute. Increasing the treatment time to 2 min the number of permeabilized cells was reduced again from 8.8 to 4.1%. The percentage of slightly permeabilized cells was reduced to 4–7% within the first treatment minutes but with increasing treatment time it continuously increased to approximately 15%. The population of intact cells remained almost constant during the treatment with 0.25% PAA with the exception of a treatment time of 1 min where only 6.1% intact cells were detected and 70.7% of the cells remained unstained. With this exception the population of unstained cells/cell fragments remained almost constant below 2% during the treatment (Figure 5A). The mean TO-fluorescence intensity of *L. innocua* significantly decreased after 0.75 min and no further significant changes were observed with increasing treatment time (Table 3). The percentage of permeabilized *L. innocua* cells increased to 61.0% during 4 min treatment with CAPP. Concurrently, the number of slightly permeabilized *L. innocua* cells increased to approximately 20% within 0.5 min and remained almost constant within the next 3.5 treatment minutes. The percentage of intact cells continuously decreased to 4.0% during CAPP treatment and the number of unstained cells/cell fragments increased to approximately 37% within the first 2.5 min and decreased again to approximately 20% within the next 1.5 min (Figure 5B). The mean TO-fluorescence intensity significantly increased due to the CAPP treatment (Table 3). The treatment with 3.42 mg l^−1^ O_3_(–) led to an increase of permeabilized *L. innocua* cells to 75.7% within 0.17 min and decreased again with increasing treatment time to 47.8% whereas the percentage of slightly permeabilized cells and the population of intact cells immediately decreased to < 1% within 0.17 min and remained constant at this level during the residual treatment time. The population of unstained cells/cell fragments continuously increased to 50.9% during ozone treatment (Figure 5C). In contrast, the population with permeabilized cells only slightly increased to approximately 99% within 0.5 min treatment with 3.8 mg l^−1^ O_3_(+) and remained almost constant at this level during the remaining treatment time. The percentage of slightly permeabilized cells and the percentage of intact cells decreased immediately to < 2% and remained at this level up to 2 min. The population of unstained cells/cell fragment continuously increased to 80–90% within 2 min (Figure 5D). *L. innocua* showed a significant decrease of mean TO-fluorescence intensity after both applied ozone treatments (Table 3).

**Table 3:** **Mean TO-fluorescence intensity of ***L. innocua*** after different treatments**.

**t [min]**	**0.25% PAA**			**3.42 mg l^−1^ O_3_(-)**			**3.8 mg l^−1^ O_3_(+)**			**20 W CAPP**		
	**Mean TO-fluorescence intensity [mV]**		***SD***	**Mean TO-fluorescence intensity [mV]**		***SD***	**Mean TO-fluorescence intensity [mV]**		***SD***	**Mean TO-fluorescence intensity [mV]**		***SD***
0	266.6^a^^,^^b^		± 69.7	1677.2^a^		± 255	436.4^a^		± 24.6	260.5^a^		± 79.6
0.17		n.d.		475.5^b^		± 210.1	211.7^b^		± 46.2		n.d.	
0.25	329.1^a^		± 70.0		n.d.			n.d.			n.d.	
0.5	169.9^b^^,^^c^		± 43.3	361.5^b^		± 77.5	273.6^b^		± 59.4	1012.8^b^		± 241.0
0.75	135.1^c^		± 10.0		n.d.			n.d.			n.d.	
1	134.8^c^		± 14.4	384.3^b^		± 166.5	214.9^b^		± 67.7	1038.9^b^		± 130.1
1.5	98.7^c^		± 9.5	237.7^b^		± 183.9	182.4^b^		± 55.6	670.9^a^^,^^b^		± 189.0
2	93.1^c^		± 20.7	257.3^b^		± 34.3	162.8^b^		± 46.2	810.9^b^		± 9.8
2.5		n.d.			n.d.			n.d.		605.7^a^^,^^b^		± 108.8
3		n.d.			n.d.			n.d.		817.4^b^		± 243.3
3.5		n.d.			n.d.			n.d.		807.7^b^		± 116.6
4		n.d.			n.d.			n.d.		755.5^b^		± 171.6

*SD, Standard deviation of three independent experiments*.

a−c *Different letters within the lines indicate significant differences at a significance level of 0.05*.

### Impact on esterase activity and membrane permeabilization

**Figure 6:**
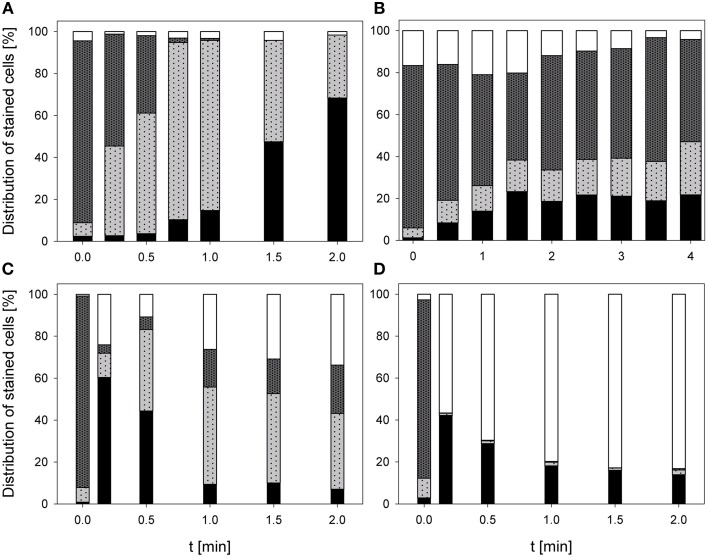
**Esterase activity and membrane integrity of ***E. coli*** after PAA treatment [(A) 10°C, 0.25% PAA], CAPP treatment [(B) 20 W], and treatment with ozonated water [(C) 3.8 mg l^−1^ O_3_(–); (D) 3.78 mg l^−1^ O_3_(+)]**. Black bars representing permeabilized cells without esterase activity; dotted gray bars representing permeabilized cells with esterase activity, dotted light gray bar representing intact cells with esterase activity, and white bars representing cells without fluorescence/cell fragments.

The treatment with 0.25% PAA led to an increase of permeabilized *E. coli* cells without esterase activity from 2.4 to 68.3% within 2 min treatment. Concurrently, intact *E. coli* cells with esterase activity decreased from 86.7 to 0%. Permeabilized cells with esterase activity increased from 6.5 to 81.2% within the first treatment minute and decreased again to 30% in the next treatment minute. The percentage of unstained cells/cell fragments remained constant < 5% during treatment time (Figure 6A). The treatment with CAPP increased the permeabilized *E. coli* cells with esterase activity to 23.3% within the first treatment minute and remained almost constant during the next 3 treatment minutes. Simultaneously, the percentage of intact cells with esterase activity decreased within 1.5 min treatment and remained almost constant at approximately 50% during the next 2.5 min. The percentage of permeabilized cells with esterase activity was continuously increasing during treatment whereas the percentage of unstained cells/cell fragment was continuously decreasing (Figure 6B). The number of permeabilized *E. coli* cells without esterase activity increased from 0.9 to 60.3% within 0.17 min treatment with 3.8 mg l^−1^ O_3_(–) and decreased again to 7.0% with increasing treatment time to 2 min. Concurrently, the percentage of intact cells with esterase activity decreased from 91.3 to 6.0% within 0.5 min and increased again to 23.1% in the next 1.5 min. Permeabilized cells with esterase activity increased from 6.4 to 46.4% within 1 min treatment and decreased again to 36.1% within the next treatment minute. The percentage of unstained cells/cell fragments is continuously increasing to 33.8% during treatment time of 2 min (Figure 6C). Similar results were obtained for the treatment with 3.8 mg l^−1^ O_3_(+) (Figure 6D). However, the percentage of unstained cells/cell fragments increased to 83.1% within 2 min and the percentage of intact cells with esterase activity immediately decreased to < 1% within 0.17 min.

**Figure 7:**
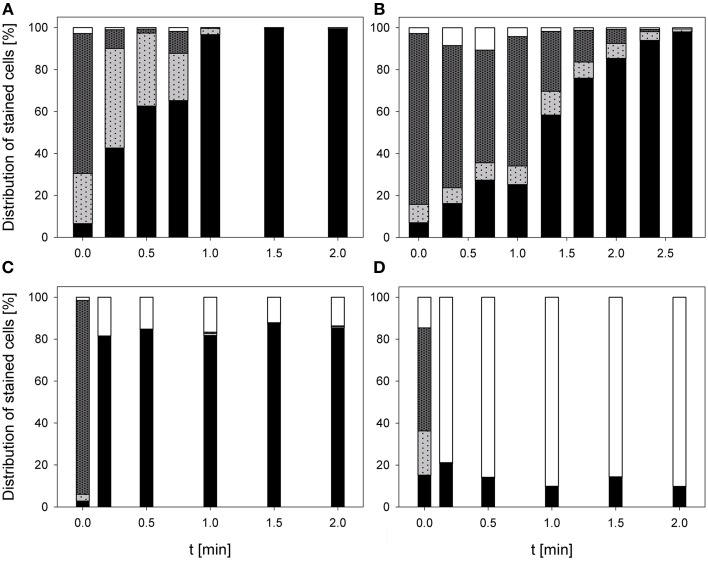
**Esterase activity and membrane integrity of ***P. carotovorum*** after PAA treatment [(A) 10°C, 0.25% PAA], CAPP treatment [(B) 20 W], and treatment with ozonated water [(C) 2.8 mg l^−1^ O_3_(–); (D) 3.8 mg l^−1^ O_3_(+)]**. Black bars representing permeabilized cells without esterase activity; dotted gray bars representing permeabilized cells with esterase activity, dotted light gray bar representing intact cells with esterase activity, and white bars representing cells without fluorescence/cell fragments.

During 2 min treatment with 0.25% PAA the percentage of permeabilized *P. carotovorum* cells without esterase activity continuously increased from 6.6% to approximately 99.2%. Simultaneously, the number of intact cells with esterase activity decreased from 66.7 to 0% and the percentage of unstained cells/cell fragments remained constant between 0 and 3%. The percentage of permeabilized cells with esterase activity increased within the first 0.25 min treatment from 23.8 to 47.4% and subsequently decreased again with increasing treatment time reaching a value below 1% (Figure 7A). The percentage of permeabilized *P. carotovorum* cells without esterase activity is continuously increasing from 7.1 to 98.0% during treatment with CAPP for 2.67 min and the percentage of intact cells with esterase activity decreased at the same time from 81.5 to 0.1%. The number of permeabilized cells with esterase activity remained almost constant within the first minute and decreased with increasing treatment time. The percentage of unstained cells/cell fragments increased in the first 0.67 min of treatment to approximately 10% and decreased to 0.5% with increasing treatment time (Figure 7B). Using an ozone concentration of 2.8 mg l^−1^ O_3_(–) increased the percentage of permeabilized cells without esterase activity from 2.8 to 81.4% within 0.17 min and with increasing treatment time the percentage of permeabilized cells without esterase activity remained almost constant. Concurrently, the number of intact cells with esterase activity decreased from 91.2 to < 1% within 0.17 min. The percentage of permeabilized cells with esterase activity remained almost constant during the ozone treatment below 3.5%. The population of unstained cells/cell fragments increased within 0.17 min to approximately 20% and remained almost constant with increasing treatment time (Figure 7C). In contrast, the percentage of permeabilized *P. carotovorum* cells without esterase activity increased from 15.2 to 20% within 0.17 min treatment with 3.8 mg l^−1^ O_3_(+) and decreased again to approximately 10% with increasing treatment time to 0.5 min and remained almost constant at this level during the next 1.5 min (Figure 7D). The percentage of intact cells with esterase activity and permeabilized cells with esterase activity immediately decreased from 49.1 to < 1% and from 21.1% to < 1%, respectively, and remained constant at this level. Simultaneously, the percentage of unstained cells/cell fragments increased to 85–90% within 0.17 min and remained almost constant at this level with increasing treatment time.

**Figure 8:**
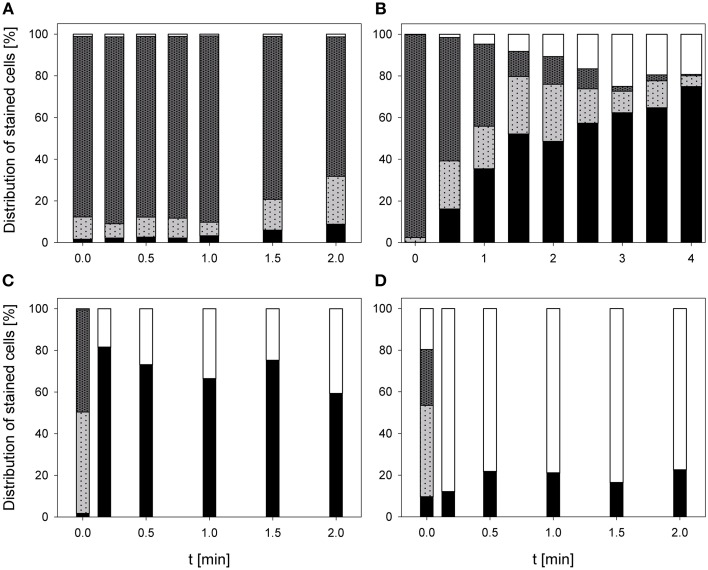
**Esterase activity and membrane integrity of ***L. innocua*** after PAA treatment [(A) 10°C, 0.25% PAA], CAPP treatment [(B) 20 W], treatment with ozonated water [(C) 3.42 mg l^−1^ O_3_(–); (D) 3.8 mg l^−1^ O_3_(+)]**. Black bars representing permeabilized cells without esterase activity; dotted gray bars representing permeabilized cells with esterase activity, dotted light gray bar representing intact cells with esterase activity, and white bars representing cells without fluorescence/cell fragments.

The number of permeabilized *L. innocua* cells without esterase activity only slightly increased to 8.8% during treatment with 0.25% PAA for 2 min. The percentage of permeabilized cells with esterase activity remained almost constant at around 10% within the first treatment minute and then increased to 23.3% within the next treatment minute. Concurrently, the percentage of intact cells with esterase activity slightly decreased from 86.6 to 66.9% within 2 min and the percentage of unstained cells/cell fragments remained almost constant at approximately 1% (Figure 8A). The treatment with CAPP led to an increase of permeabilized *L. innocua* cells without esterase activity from 0.1 to 74.9% and simultaneously, the percentage of intact cells with esterase activity decreased from 97.5 to 0.6%. The percentage of permeabilized cells with esterase activity increased within the first 2 treatment minutes from 2.3 to 27.4% and decreased again to 5.3% within the next 2 treatment minutes. The number of unstained cells/cell fragments was continuously increasing to approximately 20% (Figure 8B). Using an ozone concentration of 3.42 mg l^−1^ O_3_(–) increased the percentage of permeabilized *L. innocua* cells with esterase activity immediately within 0.17 min from 1.7 to 81.5% but with increasing treatment time the percentage decreased again to 59.3%. The percentage of permeabilized cells with esterase activity as well as the percentage of intact cells with esterase activity were also immediately reduced from 48.7 to 0% and from 49.1 to 0%, respectively, within 0.17 min but they remained constant at this level with increasing treatment time. Concurrently, the percentage of unstained cells/cell fragments continuously increased from 0.5 to 40.7% within 2 min (Figure 8C). In contrast, the percentage of permeabilized *L. innocua* cells without esterase activity slightly increased to approximately 20% during treatment with 3.8 mg l^−1^ O_3_(+) regardless of the treatment time. While the percentage of permeabilized cells with esterase activity and the percentage of intact cells with esterase activity were immediately reduced to 0% within 0.17 min, the percentage of unstained cells/cell fragments immediately increased to approximately 80% (Figure 8D).

### Mathematical modeling of inactivation kinetics and flow cytometric data

All inactivation kinetics were modeled with the log-linear regression model with tailing, the Gompertz model was used to describe the membrane permeabilization. The reduction of intact cells and the reduction of esterase activity followed a log-linear regression and the depolarization of the cells was modeled with the log-linear regression model with tailing.

Statistical measures and parameter values obtained from mathematical models fitted to the experimental data of inactivated *E. coli* are summarized in Table 4. Using 0.25% PAA at 10°C a 4 log reduction of *E. coli* could be achieved within ± 0.08 min according to the applied model and the inactivation rate was 145.6 min^−1^. The depolarization rate of *E. coli* was 34.0 min^−1^. The reduction rate of intact cells was 6.4 min^−1^ and the esterase inactivation rate of PAA treated *E. coli* cells was 92.7 min^−1^. For the cell membrane permeabilization a rate of 0.2 min^−1^ was calculated. A 4 log reduction of *E. coli* using 3.8 mg l^−1^ O_3_(–) was achieved within ± 0.1 min and the inactivation rate was 98.8 min^−1^. Depolarization of cells due to treatment with ozonated water was calculated with a rate of 101.5 min ^−1^. The decrease rate of intact cells was 58.4 min^−1^ and the cell membrane permeabilization rate was 0.01 min^−1^. The inactivation of esterase activity occurred with a rate of 33.2 min^−1^. The inactivation rate of *E. coli* was 46.5 min^−1^, the depolarization rate was 220.8 min^−1^, the decrease rate of intact cells was 55.0 min^−1^ and the rate of esterase inactivation was 54.4 min^−1^ using 3.78 mg l^−1^ O_3_(+). The cell membrane permeabilization was calculated with a rate of –0.02 min^−1^. The inactivation rate of *E. coli* after CAPP treatment was calculated as 1.3 min^−1^. The reduction of membrane potential could be achieved with a rate of 0.6 min^−1^. The decrease of intact cells was calculated with a rate of 60.5 min^−1^ while the inactivation rate of esterase was 9.4 min^−1^. Cell membrane permeabilization was calculated with a rate of 1.2 min^−1^.

**Table 4:** **Statistical measures and parameter values obtained from GInaFit Version 1.6 and SigmaPlot13 for experimental data of inactivated ***E. coli*****.

**Target site**	**Model**	**k_max_[1 min^−1^]**	**RMSE**	***R*^2^**
**0.25% PAA**				
Microbial reduction	log-linear regression + tailing	145.62±1.1^*^10^10^	0.44	0.98
Loss of membrane potential	log-linear regression + tailing	34.02±1.12	0.02	1.00
Decrease of intact cells	log-linear regression	6.35±3.02	2.26	0.47
Membrane permeabilization	Gompertz	0.23±0.67	n.s.	0.98
Decrease of esterase activity	log-linear regression	92.66±28.45	21.34	0.68
**3.8 mg l**^−1^ **O**_3_**(-)**				
Microbial reduction	log-linear regression + tailing	98.75±0.00	0.00	1.00
Loss of membrane potential	log-linear regression + tailing	101.48±21.53	1.00	1.00
Decrease of intact cells	log-linear regression	54.98±46.20	34.59	0.29
Membrane permeabilization	Gompertz	0.01±4.2^*^10^5^	n.s.	0.70
Decrease of esterase activity	log-linear regression	33.21±44.88	34.17	0.12
**3.78 mg l^−1^ O_3_(+)**				
Microbial reduction	log-linear regression + tailing	46.50±13.42	0.98	0.53
Loss of membrane potential	log-linear regression + tailing	220.83±1.4^*^10^10^	0.17	1.00
Decrease of intact cells	log-linear regression	58.44±45.43	35.18	0.26
Membrane permeabilization	Gompertz	−0.02±1.49^*^10^6^	n.s.	0.27
Decrease of esterase activity	log-linear regression	54.35±42.98	32.73	0.29
**20 W CAPP**				
Microbial reduction	log-linear regression + tailing	1.29±0.23	0.27	0.89
Loss of membrane potential	log-linear regression + tailing	0.61±1.36	0.73	0.25
Decrease of intact cells	log-linear regression	30.45±1.94	3.27	0.97
Membrane permeabilization	Gompertz	1.16±1.66	n.s.	0.94
Decrease of esterase activity	log-linear regression	9.43±5.49	9.24	0.30

*n.s., not specified*.

Statistical measures and parameter values obtained from mathematical models fitted to the experimental data of inactivated *P. carotovorum* are summarized in Table 5. A 4 log reduction of total viable count of *P. carotovorum* using PAA was achieved within ± 0.3 min and the inactivation rate was 32.2 min^−1^. The membrane depolarization rate was 72.3 min^−1^ while the reduction of intact cells was calculated with a rate of 104.5 min^−1^. The esterase inactivation rate was 49.6 min^−1^ and the cell membrane permeabilization rate was 0.01 min^−1^. A 4 log reduction of total viable count of *P. carotovorum* using 2.8 mg l^−1^ O_3_(–) was achieved within ± 0.06 min and the inactivation rate was 155.1 min^−1^. The membrane depolarization rate was 184.4 min^−1^, the decrease rate of intact cells was 53.3 min^−1^, and the esterase inactivation rate was 59.2 min^−1^. The cell membrane permeabilization was calculated with a rate of 0.05 min^−1^. The inactivation kinetic of *P. carotovorum* after treatment with 3.82 mg l^−1^ O_3_(+) showed a calculated inactivation rate of 42.86 min^−1^. The membrane depolarization could not be described with the log-linear regression model with tailing. The decrease rate of intact cells was 55.7 min^−1^. The esterase inactivation rate was 31.8 min^−1^ while the cell permeabilization rate was −2.1 min^−1^. The microbial inactivation kinetic of *P. carotovorum* by CAPP showed a 4 log reduction within ± 1 min and the inactivation rate was 9.5 min^−1^. The membrane depolarization rate was 1.6 min^−1^, the reduction of esterase activity was 76.7 min^−1^, and the reduction rate of intact cells was 73.6 min^−1^. The cell membrane permeabilization was calculated with a rate of 0.4 min^−1^.

**Table 5:** **Statistical measures and parameter values obtained from GInaFit Version 1.6 and SigmaPlot13 for experimental data of inactivated ***P. carotovorum*****.

**Target site**	**Model**	**k_max_[1 min^−1^]**	**RMSE**	***R*^2^**
**0.25% PAA**				
Microbial reduction	log-linear regression + tailing	32.19±0.60	0.05	1.00
Loss of membrane potential	log-linear regression + tailing	72.32±4.54	0.01	1.00
Decrease of intact cells	log-linear regression	104.46±15.74	11.81	0.90
Membrane permeabilization	Gompertz	0.01±4.38^*^10^5^	n.s.	0.97
Decrease of esterase activity	log-linear regression	49.61±27.64	20.73	0.39
**2.8 mg l**^−1^ **O**_3_**(-)**				
Microbial reduction	log-linear regression + tailing	155.13±3.62	0.00	1.00
Loss of membrane potential	log-linear regression + tailing	184.35±6.25^*^10^12^	0.01	1.00
Decrease of intact cells	log-linear regression	53.31±47.12	35.88	0.24
Membrane permeabilization	Gompertz	0.05±5.56	n.s.	1.00
Decrease of esterase activity	log-linear regression	59.24±46.67	35.54	0.29
**3.8 mg l^−1^ O_3_(+)**				
Microbial reduction	log-linear regression + tailing	42.86±16.88	0.82	0.86
Loss of membrane potential	log-linear regression + tailing	#	#	#
Decrease of intact cells	log-linear regression	55.69±42.25	31.17	0.30
Membrane permeabilization	Gompertz	−2.05±20.44	n.s.	0.97
Decrease of esterase activity	log-linear regression	31.78±24.79	18.88	0.29
**20 W CAPP**				
Microbial reduction	log-linear regression + tailing	9.5±0.93	0.69	0.94
Loss of membrane potential	log-linear regression + tailing	1.61±0.73	0.0975	0.8178
Decrease of intact cells	log-linear regression	73.57±10.53	11.61	0.88
Membrane permeabilization	Gompertz	0.39±0.12	n.s.	0.98
Decrease of esterase activity	log-linear regression	76.72±7.53	8.45	0.93

*^#^Kinetic could not be described with the applied model*.

*n.s., not specified*.

Statistical measures and parameter values obtained from mathematical models for experimental data of inactivated *L. innocua* are summarized in Table 6. The microbial inactivation of *L. innocua* after treatment with 0.25% PAA could not be described with the applied model because the total viable count increased again after 1 min treatment time. The depolarization rate was 118.5 min^−1^, the decrease rate of intact cells was 50.1 min^−1^, and esterase inactivation rate was 22.8 min^−1^. The cell membrane permeabilization rate was 0.2 min^−1^. A 4 log reduction of total viable count of *L. innocua* after treatment with 3.42 mg l^−1^ O_3_(–) was achieved within ± 0.06 min and the inactivation rate was 212.4 min^−1^. The loss of membrane potential was calculated with a rate of 14.3 min^−1^. The decrease rate of intact cells was 54.1 min^−1^ and the esterase inactivation rate was 31.7 min^−1^. Using 3.8 mg l^−1^ O_3_(+), the inactivation rate of *L. innocua* was 47.8 min^−1^, the cell membrane permeabilization and the membrane depolarization of *L. innocua* was calculated with a rate of –0.2 and 4.5 min^−1^, respectively. The decrease rate of intact cells was 30.2 min^−1^ and the inactivation rate of esterase was 17.3 min^−1^. The cell membrane permeabilization was calculated with a rate of −0.20 min^−1^. Within ± 2.92 min a 4 log reduction of total viable count of *L. innocua* could be realized by CAPP treatment and the inactivation rate was 3.3 min^−1^. The depolarization rate due to CAPP treatment was 57.6 min^−1^. The decrease of intact cells was calculated with a rate of 42.6 min^−1^ and the esterase inactivation rate was 48.6 min^−1^. The cell permeabilization was calculated with a rate of 0.5 min^−1^.

**Table 6:** **Statistical measures and parameter values obtained from GInaFit Version 1.6 and SigmaPlot13 for experimental data of inactivated ***L. innocua*****.

**Target site**	**Model**	**k_max_[1 min^−1^]**	**RMSE**	***R*^2^**
**0.25% PAA**				
Microbial reduction	log-linear regression + tailing	#	#	#
Loss of membrane potential	log-linear regression + tailing	118.52±1.62	0.12	1.00
Decrease of intact cells	log-linear regression	50.06±19.88	14.91	0.56
Membrane permeabilization	Gompertz	0.15±2.33^*^10^6^	n.s.	0.98
Decrease of esterase activity	log-linear regression	22.80±6.50	4.88	0.71
**3.42 mg l**^−1^ **O**_3_**(-)**				
Microbial reduction	log-linear regression + tailing	212.36±1.20^*^10^10^	0.35	0.99
Loss of membrane potential	log-linear regression + tailing	14.31±7.4	0.08	0.91
Decrease of intact cells	log-linear regression	54.10±42.31	32.22	0.29
Membrane permeabilization	Gompertz	−0.21±6.04^*^10^6^	n.s.	0.13
Decrease of esterase activity	log-linear regression	31.69±24.8	18.89	0.29
**3.8 mg l^−1^ O_3_(+)**				
Microbial reduction	log-linear regression + tailing	47.79±9.72	0.30	0.97
Loss of membrane potential	log-linear regression + tailing	4.53±11.49	2.13	0.18
Decrease of intact cells	log-linear regression	30.15±23.49	17.89	0.29
Membrane permeabilization	Gompertz	−0.20± inf	n.s.	0.93
Decrease of esterase activity	log-linear regression	17.34±13.56	10.33	0.29
**20 W CAPP**				
Microbial reduction	log-linear regression + tailing	3.3±1.46	0.44	0.98
Loss of membrane potential	log-linear regression + tailing	57.58±2.19^*^10^9^	0.05	1.00
Decrease of intact cells	log-linear regression	42.63±7.82	13.16	0.81
Membrane permeabilization	Gompertz	0.45±0.18	n.s.	0.99
Decrease of esterase activity	log-linear regression	48.60±10.22	17.95	0.73

*^#^Kinetic could not be described with the applied model*.

*n.s., not specified*.

## Discussion

It is assumed that PAA induces oxidation of proteins, enzymes, or other metabolites and acts on DNA bases (Kitis, 2004). In order to investigate the inactivation mechanism of PAA on bacteria flow cytometric analyses were performed. Although the flow cytometric analysis suggests that most bacteria cells were intact after 0.25 min PAA treatment only a small number of colony forming units were detectable by a plate count method. This implies cell permeabilization is not the cause of the loss of culturability and other mechanisms must induce this effect. Rossi et al. (2007) found a constant decrease in viable heterotrophic bacteria using SYBR Green I and PI. But the percentage of intact cells was about 50% even after 36 min PAA treatment. The percentage of damaged cells (double-stained cells with SYBR Green I + PI) remained constant during 5 mg l^−1^ PAA treatment. Similar results were obtained for total heterotrophic bacteria during 5 mg l^−1^ PAA treatment (Antonelli et al., 2006) and 15 and 25 mg l^−1^ PAA treatment (Mezzanotte et al., 2003). Increasing mean TO-fluorescence intensity of cells indicates that TO is bound to DNA and not to RNA (Nygren et al., 1998). The increase of mean TO-fluorescence intensity implies that RNA was damaged due to PAA treatment and TO was predominantly bound to DNA. The decrease of mean TO-fluorescence intensity with increasing treatment time can be explained by the increased amount of PI in the cells that quenched the TO-fluorescence. The loss of culturability after 0.25 min PAA treatment at different temperatures is evidently not related to cell death. According to Bunthof (2002) the physiological status of cells can be classified in four states with culturability as highest form of physiological fitness. Nevertheless, the cells that lost culturability might be able to recover and subsequently cause disease. After PAA treatment the red/green DiOC_2_(3) ratio was reduced to approximately 1.0 indicating a reduced membrane potential of all tested bacteria. However, *E. coli* cells are not completely depolarized because red/green ratio of completely depolarized cells is below one. The membrane potential is involved in numerous processes of bacterial physiology and is strongly related to bacterial viability (Novo et al., 1999). The measurement of a low membrane potential after PAA treatment supports the assumption that the cells may still cause diseases. Cells still showed esterase activity after PAA treatment, this indicates that the cells still have metabolic activity but according to the plate count results these cells were no longer culturable. Increasing treatment times showed lower cF-fluorescence intensity as untreated bacteria cells. This indicates that a lower amount of cFDA was hydrolyzed after PAA treatment which may be related to a lower amount of active esterase within the cells. This may be a result of an oxidation of enzymes by PAA (Kitis, 2004). Park et al. (2014) found a higher reduction of *Enterococcus faecium* using 5 ppm PAA measured by plate count than by the ATP bioluminescence method. They suggested that the bacteria are injured due to the PAA treatment but they are still viable. Modeling of changes in bacterial physiological properties measured by flow cytometry after PAA treatment may help to determine the most sensitive target sites of bacteria to PAA.

The inactivation of bacteria by ozone is attributed to changes in cell permeability and cell lyses (Moore et al., 2000) which can elicit the changes in scatter intensities. Consequently, the membrane integrity of bacteria cells after ozone treatment was investigated. Ozone leads to a rupture of cells and cell lyses with subsequent attack on nucleic acids and enzymes (Pascual et al., 2007). The low fluorescence intensity of TO can be related to unbound dye adherent to cell fragments and the occurrence of PI-fluorescence indicates that this cell population did not show cell lyses. The unstained fraction indicates that the cells are highly permeabilized so that cell lyses occurred and therefore, no fluorescence could be detected. It can also be assumed that nucleic acids are affected by the ozone and therefore, an intercalating of fluorescent dyes with DNA and RNA was not possible. A complete destruction of bacteria in drinking water after ozonation was observed using SYBR Green I and propidium iodide. Along with a few detectable bacteria cells a high amount of cell debris was detected with the flow cytometer after ozonation of drinking water (Hammes et al., 2008). Similar results were obtained for bacteria cells from ozonated river water after staining with SYBR Green and PI. The PI-labeled fraction was not able to form colonies on agar plates (Grégori et al., 2001). The predominant fraction of *L. innocua* and *P. carotovorum* cells is permeabilized indicating that these cells were not completely destroyed by the ozone treatment. However, the unstained fraction of *L. innocua* cells was higher than the unstained fraction of *P. carotovorum* which lead to the assumption that a much greater number of the population of *L. innocua* was destroyed completely. The observed damage of Gram-negative and Gram-positive bacteria cells is consistent with the observations of Thanomsub et al. (2002). According to the literature, Gram-Positive bacteria are more sensitive to ozone than Gram-negative bacteria (Tiwari, 2012). In our study, the results of the plate count methods showed the highest sensitivity of *P. carotovorum* against ozone while the highest amount of intact cells was observed by flow cytometry. Thus, the loss of culturability of *P. carotovorum* cells is not directly correlated with the loss of membrane integrity. Different results were obtained after ozone treatment stopped by Na_2_S_2_O_3_ in comparison to the results obtained after ozone treatment stopped by shaking. For all tested bacteria a high amount of unstained cells were detected after ozone treatment. Again, *P. carotovorum* showed the highest percentage of intact cells and *L. innocua* the lowest percentage of intact cells. The high number of unstained cells suggests a complete destruction of bacteria cells after ozone treatment. This is inconsistent with the results obtained by plate count method because a high number of viable bacteria were detected while flow cytometric analyses of membrane integrity suggest complete destruction of cells. This implies that the unstained fraction of cells cannot necessarily be related to destroyed cells. It is also possible that a flocculation of cellular proteins and an oxidation of double-bonds (Kim et al., 1999) restricted the dye uptake without affecting the culturability. Zhang et al. (2011) showed that *Pseudomonas aeruginosa* remained intact after treatment with ozone and they suggested that the inactivation is due to increased cytoplasmic membrane permeability and cytoplasm coagulation. Alternatively, the unstained cells may have been completely destroyed cells and cell lyses occurred while the permeabilized fraction may be able to form colonies on a medium because permeabilization of cells is reversible. A reversible pore formation has been described for bacteria treated with pulsed electric fields (Garcia et al., 2007). While the ozone improved cell permeability and enabled the uptake of PI, the availability of nutrients in terms of Na_2_S_2_O_3_ can be metabolically used to support the growth of bacteria on a cultivating medium. These results clearly indicate that membrane integrity measurements cannot give reliable prediction of cell viability. A proposed cause of bacterial death due to ozone treatment is the oxidation of sulfhydryl groups in enzymes (Kim et al., 1999). However, the cF-fluorescence intensity of bacteria is very low after ozone treatment indicating a small amount of hydrolyzed cFDA due to an inactivation of esterase. This supports the assumption that the esterase is oxidized by ozone. Alternatively, cFDA may be hydrolyzed by esterase but due to the permeabilization of cells cF cannot be retained within the cells. The centrifugation step to remove surplus cFDA may also remove the hydrolyze product cF which could lead to an underestimation of esterase activity. But on the other hand the percentage of permeabilized cells with esterase activity increased. This leads to the assumption that the cell membrane is affected by the ozone before the esterase is affected. It has been shown that a short-time exposure of *E. coli* to ozone led to membrane damage while a long-time exposure affects the intracellular components (Komanapalli and Lau, 1996). Esterase activity of bacteria was also tested after ozone treatment and quenching of ozone by Na_2_S_2_O_3_. No esterase activity was observed after 0.17 min ozone treatment for all tested bacteria. Similar to the results obtained by the measurement of membrane integrity, the predominant fraction of cells remained unstained. This implies that the unstained fraction represents completely ruptured cells and that cell lyses occurred. However, the number of culturable bacteria is not neglectable and this contradicts with the presence of ruptured cells. Therefore, it is more likely that cell permeabilization measured by PI uptake is reversible and cells were able to form colonies on agar medium in the presence of nutrient. The oxidation of phospholipids and proteins in cell membranes is shown to alter the regulation of cell permeability without affecting cell permeability (Cronan and Vagelos, 1972). Thus, the uptake of dyes may be prevented resulting in a high percentage of unstained cells. The generation of membrane potential by tested bacteria was restricted after ozone treatment at different concentrations but the red/green DiOC_2_(3) ratio was > 1 indicating that the cells were not completely depolarized after ozone treatment. The depolarization of the cells increased with increasing ozone concentration. However, the highest membrane potential was observed for bacteria cells after ozone treatment (3.8 mg l^−1^) stopped by the addition of sodium thiosulfate. This implies that the cells are still able to generate membrane potential after ozone treatment although the application of TO/PI and cFDA/PI did not clearly indicate cell viability. It is not possible to distinguish which cells were able to generate membrane potential after ozone treatment because the measurement represents the average of membrane potential of a population and not the membrane potential of single cells. According to Davidson and Branden (1981) the mode of microbial inactivation of ozone is categorized into three groups: (i) cell membrane is permeabilized and loss of cellular compounds occurs; (ii) inactivation of essential enzymes; and (iii) destruction or functional inactivation of genetic material. After ozone treatment the application of fluorescent dyes did not clearly differentiate between viable, sublethally damaged, and lethally damaged cells. A high fraction of unstained cells restricted the interpretation of physiological properties. Nevertheless, the obtained data were modeled in order to compare the different inactivation treatments and to describe the mode of action of ozone.

To investigate the inactivation mechanism of CAPP on *E. coli, L. innocua*, and *P. carotovorum* inoculated on a polysaccharide gel flow cytometric analyses were performed after treatment. In contrast to previous experiments (Fröhling et al., 2012a), *L. innocua* was more sensitive to CAPP treatment than *E. coli*. A possible explanation can be the different growth conditions of *L. innocua* in both studies which implies that the success of inactivation is highly dependent on the physiological status of the bacteria. *E. coli* was also more resistant against CAPP treatment than *P. carotovorum*. Similar to the treatment with ozonated water the CAPP showed different mode of actions against the tested bacteria whereas the greatest differences were detected between Gram-negative and Gram-positive bacteria. Different sensitivity of bacteria was also found by Baier et al. (2015). They showed that CAPP treatment has a different impact on three *E. coli* serovars tested. At 20 W the percentage of intact *L. innocua* cells decreased but the percentage of permeabilized cells did not increase proportionally. The large amount of unstained cells/cell fragments may indicate a complete rupture of the cell membrane and associated cell lyses. Another explanation for the unstained cells/cell fragments is that the RNA and DNA were affected by the treatment. This assumption is supported by the significant increase in mean TO fluorescence intensity during plasma treatment. The plasma induced inactivation of *Citrobacter freundii* in apple juice is based on the cell permeabilization due to degradation of specific proteins and lipids. This cell permeabilization may enable the reactive compounds to penetrate into the cells and damage the RNA and possibly the DNA (Surowsky et al., 2014a). However, it is not clear whether the RNA and DNA damage is caused by UV radiation or due to oxidation by oxygen radicals as both of these have been suggested as possible inactivation mechanisms for high pressure plasma. Previous experiments showed that the UV irradiation generated by a similar plasma jet had minor influence on the bacteria (Brandenburg et al., 2007). However, argon supported the UV transmission and UV can support the inactivation. In contrast, the percentage of unstained Gram-negative bacteria remained almost constant after plasma treatment. The decrease of TO-intensity with increasing treatment time may be the result of enhanced membrane permeabilization resulting in competitive intercalating of PI and TO with DNA and quenching of TO-fluorescence by the PI-fluorescence.

Modeling of the flow cytometric data showed a different sensitivity of the bacteria to the applied treatment and that different target sites of the bacteria were affected by the treatments. The loss of culturability of *E. coli* was faster after PAA and O_3_ treatment when compared to CAPP treatment whereas loss of culturability of *P. carotovorum* and *L. innocua* first occurred after ozone treatment followed by PAA and CAPP treatment. The membrane depolarization of *E. coli* and *P. carotovorum* was faster after ozone treatment followed by PAA treatment and CAPP treatment. In contrast, membrane depolarization first occurred for the PAA treatment of *L. innocua* occurred followed by CAPP and ozone treatment. Cell membrane permeabilization of *E. coli* and *L. innocua* occurred faster after CAPP treatment followed by PAA treatment and ozone treatment. Similar results were obtained for the cell membrane permeabilization of *P. carotovorum* with the exception that the permeabilization occurred quicker using O_3_(–) than PAA treatment. The esterase activity of *L. innocua* and *P. carotovorum* was faster affected using CAPP treatment followed by O_3_(–), PAA, and then O_3_(+) treatment whereas esterase of *E. coli* was faster inactivated by PAA followed by ozone treatment and finally CAPP treatment. Due to the high percentage of unstained cells and double-stained cells which were not considered in the modeling an overestimation or underestimation of the rates of physiological changes may be derived. It could not be verified if the unstained fraction after the treatments corresponds to complete ruptured cells or to cells that still have intact physiological properties, but which were not detected with the applied fluorescent dyes due to restricted dye uptake. It had to be taken into account that double-stained cells representing on one hand slightly permeabilized cells, and on the other hand permeabilized cells with esterase activity were not considered in the mathematical models. This may lead to an underestimation of cell permeabilization and overestimation of the reduction of esterase activity.

The inactivation rate for CAPP treatment is longer when compared with PAA and ozone treatment. Although, it should be noted that the comparison of PAA, ozone, and CAPP treatment was restricted, bacteria cells were treated with PAA and ozone in suspension whereas bacteria cells treated with CAPP were adherent on a surface (because of the formation of different bacteria layers on the surface of the gel, the superficial bacteria layer may have protective effects against the lowest bacteria layer resulting in reduced inactivation). However, the CAPP treatment procedure is more related to food surface decontamination than the other tested treatment procedures. The use of flow cytometric measurements to characterize different inactivation treatments enables the examination of inactivation effects at a single cell level within a short time. With the applied fluorescent dyes it was possible to evaluate the membrane integrity, esterase activity, pump activity, and membrane potential of Gram-negative and Gram-positive bacteria after the different inactivation treatments used in this study. It was shown that the bacteria were not homogeneously inactivated by the treatments tested because different cell populations were detected by flow cytometry. The reliability of measurements is dependent on the inactivation processes applied. In case of complete membrane rupture and cell lyses the application of fluorescent dyes seems to be restricted because a high amount of undefined fluorescence is detected. Furthermore, as a result of the damage of intracellular components the applied fluorescent dyes could not intercalate or could not be metabolized. This also leads to a high amount of unstained cells which may result in an overestimation or underestimation of inactivation effects. Nevertheless, it was shown that flow cytometric measurements provide important information of bacteria cell status after different inactivation treatments within a short time. In a first approach, the membrane integrity was modeled with the Gompertz model or a logistic and esterase activity and membrane potential were modeled with the GInaFiT tool. Thus, the determination of permeabilization, depolarization, and the esterase inactivation rates was possible and facilitates the comparison of different inactivation processes as well as the prediction of inactivation effects. However, the kinetics of unstained cells and double-stained cells are not considered in the modeling. Thus, important information regarding the inactivation mechanisms could have been lost. It was not possible to model the obtained curves for unstained cells and double-stained cells with common available mathematical models due to the irregular shape of the curves. This may lead to a misinterpretation of inactivation mechanisms. However, the modeling of physiological property changes obtained by flow cytometric measurements can help to predict inactivation kinetics. The detailed knowledge of inactivation effects is absolutely necessary for the implementation of inactivation processes in the production chain.

### Conflict of interest statement

The authors declare that the research was conducted in the absence of any commercial or financial relationships that could be construed as a potential conflict of interest.

## References

[B1] Alvaro J. E. Moreno S. Dianez F. Santos M. Carrasco G. Urrestarazu M. (2009). Effects of peracetic acid disinfectant on the postharvest of some fresh vegetables. J. Food Eng. 95, 11–15. 10.1016/j.jfoodeng.2009.05.003

[B2] Ananta E. Knorr D. (2009). Comparison of inactivation pathways of thermal or high pressure inactivated *Lactobacillus rhamnosus* ATCC 53103 by flow cytometry analysis. Food Microbiol. 26, 542–546. 10.1016/j.fm.2009.01.008 19465252

[B3] Ananta E. Voigt D. Zenker M. Heinz V. Knorr D. (2005). Cellular injuries upon exposure of *Escherichia coli* and *Lactobacillus rhamnosus* to high-intensity ultrasound. J. Appl. Microbiol. 99, 271–278. 10.1111/j.1365-2672.2005.02619.x 16033457

[B4] Antonelli M. Rossi S. Mezzanotte V. Nurizzo C. (2006). Secondary effluent disinfection: PAA long term efficiency. Environ. Sci. Technol. 40, 4771–4775. 10.1021/es060273f 16913137

[B5] Baier M. Janßen T. Wieler L. H. Ehlbeck J. Knorr D. Schlüter O. (2015). Inactivation of Shiga toxin-producing *Escherichia coli* O104:H4 using cold atmospheric pressure plasma. J. Biosci. Bioeng. 120, 275–279. 10.1016/j.jbiosc.2015.01.003 25782617

[B6] Berney M. Hammes F. Bosshard F. Weilenmann H. U. Egli T. (2007). Assessment and interpretation of bacterial viability by using the LIVE/DEAD BacLight kit in combination with flow cytometry. Appl. Environ. Microbiol. 73, 3283–3290. 10.1128/AEM.02750-06 17384309 PMC1907116

[B7] Beuchat L. R. (1998). Surface Decontamination of Fruits and Vegetables Eaten Raw: A Review. WHO/FSF/FOS/98.2. Geneva: World Health Organization.

[B8] Beuchat L. R. Adler B. B. Lang M. M. (2004). Efficacy of chlorine and a peroxyacetic acid sanitizer in killing *Listeria monocytogenes* on iceberg and romaine lettuce using simulated commercial processing conditions. J. Food Prot. 67, 1238–1242. 15222557 10.4315/0362-028x-67.6.1238

[B9] Bigoni R. Kötzsch S. Sorlini S. Egli T. (2014). Solar water disinfection by a Parabolic Trough Concentrator (PTC): flow-cytometric analysis of bacterial inactivation. J. Clean. Prod. 67, 62–71. 10.1016/j.jclepro.2013.12.014

[B10] Boudam M. K. Moisan M. Saoudi B. Popovici C. Gherardi N. Massines F. (2006). Bacterial spore inactivation by atmospheric-pressure plasmas in the presence or absence of UV photons as obtained with the same gas mixture. J. Phys. D Appl. Phys. 39, 3494–3507. 10.1088/0022-3727/39/16/S07

[B11] Brandenburg R. Ehlbeck J. Stieber M. Von Woedtke T. Zeymer J. Schlüter O. (2007). Antimicrobial treatment of heat sensitive materials by means of atmospheric pressure rf-driven plasma jet. Contrib. Plasma Phys. 47, 72–79. 10.1002/ctpp.200710011

[B12] Bunthof C. J. (2002). Flow Cytometry, Fluorescent Probes, and Flashing Bacteria. Wageningen: Wageningen University.

[B13] Bunthof C. J. Abee T. (2002). Development of a flow cytometric method to analyze subpopulations of bacteria in probiotic products and dairy starters. Appl. Environ. Microbiol. 68, 2934–2942. 10.1128/AEM.68.6.2934-2942.2002 12039752 PMC123932

[B14] Bußler S. Herppich W. B. Neugart S. Schreiner M. Ehlbeck J. Rohn S. (2015a). Impact of cold atmospheric pressure plasma on physiology and flavonol glycoside profile of peas (*Pisum sativum ‘Salamanca’*). Food Res. Int. 76, 132–141. 10.1016/j.foodres.2015.03.045

[B15] Bußler S. Steins V. Ehlbeck J. Schlüter O. (2015b). Impact of thermal treatment versus cold atmospheric plasma processing on the techno-functional protein properties from *Pisum sativum ‘Salamanca’*. J. Food Eng. 10.1016/j.jfoodeng.2015.05.036 [Epub ahead of print].

[B16] Cronan J. E. Vagelos P. R. (1972). Metabolism and function of the membrane phospholipids of *Escherichia coli*. Biochim. Biophys. Acta 265, 25–60. 10.1016/0304-4157(72)90018-4 4552305

[B17] Da Silveira M. G. Abee T. (2009). Activity of ethanol-stressed *Oenococcus oeni* cells: a flow cytometric approach. J. Appl. Microbiol. 106, 1690–1696. 10.1111/j.1365-2672.2008.04136.x 19226398

[B18] Davidson P. M. Branden A. L. (1981). Anti-microbial activity of non-halogenated phenolic-compounds. J. Food Protect. 44, 623–632. 10.4315/0362-028X-44.8.623 30836539

[B19] Doyle M. P. Erickson M. C. (2008). Summer meeting 2007 - the problems with fresh produce: an overview. J. Appl. Microbiol. 105, 317–330. 10.1111/j.1365-2672.2008.03746.x 18284485

[B20] Ehlbeck J. Schnabel U. Polak M. Winter J. Woetke T. V. Brandenburg R. (2011). Low temperature atmospheric pressure plasma sources for microbial decontamination. J. Phys. D Appl. Phys. 44:013002 10.1088/0022-3727/44/1/013002

[B21] El Shafie A. Fouda M. M. G. Hashem M. (2009). One-step process for bio-scouring and peracetic acid bleaching of cotton fabric. Carbohydr. Polym. 78, 302–308. 10.1016/j.carbpol.2009.04.002

[B22] Fröhling A. Baier M. Ehlbeck J. Knorr D. Schlüter O. (2012a). Atmospheric pressure plasma treatment of *Listeria innocua* and *Escherichia coli* at polysaccharide surfaces: inactivation kinetics and flow cytometric characterization. Innovative Food Sci. Emerg. Technol. 13, 142–150. 10.1016/j.ifset.2011.11.002

[B23] Fröhling A. Klocke S. Hausdorf L. Klocke M. Schlüter O. (2012b). A method for viability testing of *Pectobacterium carotovorum* in postharvest processing by means of flow cytometry. Food Bioprocess Technol. 5, 2871–2879. 10.1007/s11947-011-0749-6

[B24] García D. Gómez N. Mañas P. Raso J. Pagán R. (2007). Pulsed electric fields cause bacterial envelopes permeabilization depending on the treatment intensity, the treatment medium pH and the microorganism investigated. Int. J. Food Microbiol. 113, 219–227. 10.1016/j.ijfoodmicro.2006.07.007 16987561

[B25] Gaunt L. F. Beggs C. B. Georghiou G. E. (2006). Bactericidal action of the reactive species produced by gas-discharge nonthermal plasma at atmospheric pressure: a review. IEEE Trans. Plasma Sci. 34, 1257–1269. 10.1109/TPS.2006.878381

[B26] Geeraerd A. H. Valdramidis V. P. Van Impe J. F. (2005). GInaFiT, a freeware tool to assess non-log-linear microbial survivor curves. Int. J. Food Microbiol. 102, 95–105. 10.1016/j.ijfoodmicro.2004.11.038 15893399

[B27] Graham D. (1997). Use of ozone for food processing. Food Technol. 51, 72–76.

[B28] Grégori G. Citterio S. Ghiani A. Labra M. Sgorbati S. Brown S.. (2001). Resolution of viable and membrane-compromised bacteria in freshwater and marine waters based on analytical flow cytometry and nucleic acid double staining. Appl. Environ. Microbiol. 67, 4662–4670. 10.1128/AEM.67.10.4662-4670.2001 11571170 PMC93217

[B29] Guzel-Seydim Z. Bever P. Greene A. (2004). Efficacy of ozone to reduce bacterial populations in the presence of food components. Food Microbiol. 21, 475–479. 10.1016/j.fm.2003.10.001

[B30] Hammes F. Berney M. Wang Y. Y. Vital M. Köster O. Egli T. (2008). Flow-cytometric total bacterial cell counts as a descriptive microbiological parameter for drinking water treatment processes. Water Res. 42, 269–277. 10.1016/j.watres.2007.07.009 17659762

[B31] Haugland R. P. (1994). Spectra of fluorescent dyes used in flow cytometry. Methods Cell Biol. 42, 641–663. 7533258 10.1016/s0091-679x(08)61100-0

[B32] Herrero M. Quirós C. García L. A. Díaz M. (2006). Use of flow cytometry to follow the physiological states of microorganisms in cider fermentation processes. Appl. Environ. Microbiol. 72, 6725–6733. 10.1128/AEM.01183-06 17021224 PMC1610271

[B33] Hewitt C. J. Nebe-Von-Caron G. (2004). The application of multi-parameter flow cytometry to monitor individual microbial cell physiological status. Adv. Biochem. Eng. Biotechnol. 89, 197–223. 10.1007/b93997 15217160

[B34] Johnson S. Nguyen V. Coder D. (2013). Assessment of cell viability. Curr. Protoc. Cytom. 64:9.2.1–9.2.26. 10.1002/0471142956.cy0902s64 23546778

[B35] Joux F. Lebaron P. (2000). Use of fluorescent probes to assess physiological functions of bacteria at single-cell level. Microb. Infect. 2, 1523–1535. 10.1016/S1286-4579(00)01307-1 11099939

[B36] Joyce E. Al-Hashimi A. Mason T. J. (2011). Assessing the effect of different ultrasonic frequencies on bacterial viability using flow cytometry. J. Appl. Microbiol. 110, 862–870. 10.1111/j.1365-2672.2011.04923.x 21324052

[B37] Kader A. A. (2005). Increasing food availability by reducing postharvest losses of fresh produce. Acta Hortic. (ISHS) 682, 2169–2176. 10.17660/actahortic.2005.682.296

[B38] Kamat A. S. Nair P. M. (1996). Identification of *Listeria innocua* as a biological indicator for inactivation of *L. monocytogenes* by some meat processing treatments. Lebensm. Wiss. Technol. 29, 714–720. 10.1006/fstl.1996.0111

[B39] Khadre M. Yousef A. E. (2001). Sporicidal action of ozone and hydrogen peroxide: a comparative study. Int. J. Food Microbiol. 71, 131–138. 10.1016/S0168-1605(01)00561-X 11789930

[B40] Kim H. Ryu J. H. Beuchat L. R. (2006). Survival of *Enterobacter sakazakii* on fresh produce as affected by temperature, and effectiveness of sanitizers for its elimination. Int. J. Food Microbiol. 111, 134–143. 10.1016/j.ijfoodmicro.2006.05.021 16891023

[B41] Kim J.-G. Yousef A. E. Dave S. (1999). Application of ozone for enhancing the microbiological safety and quality of foods: a review. J. Food Prot. 62, 1071–1087. 10492485 10.4315/0362-028x-62.9.1071

[B42] Kim J.-G. Yousef A. E, Khadre, M. A. (2003). Ozone and its current and future application in the food industry. Adv. Food Nutr. Res. 45, 167–218. 10.1016/S1043-4526(03)45005-5 12402681

[B43] Kitis M. (2004). Disinfection of wastewater with peracetic acid: a review. Environ. Int. 30, 47–55. 10.1016/S0160-4120(03)00147-8 14664864

[B44] Koivunen J. Heinonen-Tanski H. (2005). Peracetic acid (PAA) disinfection of primary, secondary and tertiary treated municipal wastewaters. Water Res. 39, 4445–4453. 10.1016/j.watres.2005.08.016 16221481

[B45] Komanapalli I. R. Lau B. H. S. (1996). Ozone-induced damage of *Escherichia coli* K-12. Appl. Microbiol. Biotechnol. 46, 610–614. 10.1007/s002530050869 9008892

[B46] Kunigk L. Almeida M. C. B. (2001). Action of peracetic acid on *Escherichia coli* and *Staphylococcus aureus* in suspension and on stainless steel surfaces. Braz. J. Microbiol. 32, 38–41. 10.1590/S1517-83822001000100009

[B47] Laroussi M. (2002). Nonthermal decontamination of biological media by atmospheric-pressure plasmas: review, analysis and prospects. IEEE Trans. Plasma Sci. 30, 1409–1415. 10.1109/TPS.2002.804220

[B48] Laroussi M. (2005). Low temperature plasma-based sterilization: overview and state-of-the-art. Plasma Processes Polym. 2, 391–400. 10.1002/ppap.200400078

[B49] Luscher C. Balasa A. Fröhling A. Ananta E. Knorr D. (2004). Effect of high-pressure-induced ice I-to-ice III phase transitions on inactivation of *Listeria innocua* in frozen suspension. Appl. Environ. Microbiol. 70, 4021–4029. 10.1128/AEM.70.7.4021-4029.2004 15240278 PMC444759

[B50] Luukkonen T. Teeriniemi J. Prokkola H. Rämö J. Lassi U. (2014). Chemical aspects of peracetic acid based wastewater disinfection. Water SA 40, 73–80. 10.4314/wsa.v40i1.9

[B51] Mahapatra A. K. Muthukumarappan K. Julson J. (2005). Applications of ozone, bacteriocins and irradiation in food processing: a review. Crit. Rev. Food Sci. Nutr. 45, 447–461. 10.1080/10408390591034454 16183567

[B52] Mathys A. Chapman B. Bull M. Heinz V. Knorr D. (2007). Flow cytometric assessment of *Bacillus* spore response to high pressure and heat. Innovative Food Sci. Emerg. Technol. 8, 519–527. 10.1016/j.ifset.2007.06.010

[B53] Mezzanotte V. Antonelli M. Azzellino A. Citterio S. Nurizzo C. (2003). Secondary effluent disinfection by peracetic acid (PAA): microrganism inactivation and regrowth, preliminary results. Water Sci. Technol. 3, 269–275.

[B54] Miller F. A. Silva C. L. M. Brandao T. R. S. (2013). A review on ozone-based treatments for fruit and vegetables preservation. Food Eng. Rev. 5, 77–106. 10.1007/s12393-013-9064-5

[B55] Misra N. N. Kaur S. Tiwari B. K. Kaur A. Singh N. Cullen P. J. (2015). Atmospheric pressure cold plasma (ACP) treatment of wheat flour. Food Hydrocoll. 44, 115–121. 10.1016/j.foodhyd.2014.08.019

[B56] Moisan M. Barbeau J. Crevier M.-C. Pelletier J. Philip N. Saoudi B. (2002). Plasma sterilization. Methods and mechanisms. Pure Appl. Chem. 74, 349–358. 10.1351/pac200274030349

[B57] Moisan M. Barbeau J. Moreau S. Pelletier J. Tabrizian M. Yahia L. H. (2001). Low-temperature sterilization using gas plasmas: a review of the experiments and an analysis of the inactivation mechanisms. Int. J. Pharm. 226, 1–21. 10.1016/S0378-5173(01)00752-9 11532565

[B58] Moore G. Griffith C. Peters A. (2000). Bactericidal properties of ozone and its potential application as a terminal disinfectant. J. Food Prot. 63, 1100–1106. 10945587 10.4315/0362-028x-63.8.1100

[B59] Moreau M. Orange N. Feuilloley M. G. J. (2008). Non-thermal plasma technologies: new tools for bio-decontamination. Biotechnol. Adv. 26, 610–617. 10.1016/j.biotechadv.2008.08.001 18775485

[B60] Novo D. Perlmutter N. G. Hunt R. H. Shapiro H. M. (1999). Accurate flow cytometric membrane potential measurement in bacteria using diethyloxacarbocyanine and a ratiometric technique. Cytometry 35, 55–63. 10554181 10.1002/(sici)1097-0320(19990101)35:1<55::aid-cyto8>3.0.co;2-2

[B61] Nygren J. Svanvik N. Kubista M. (1998). The interactions between the fluorescent dye thiazole orange and DNA. Biopolymers 46, 39–51. 9612138 10.1002/(SICI)1097-0282(199807)46:1<39::AID-BIP4>3.0.CO;2-Z

[B62] O'Donnell C. Tiwari B. K. Cullen P. J. Rice R. G. (2012). Ozone in Food Processing. West Sussex: Wiley-Blackwell.

[B63] Ölmez H. Kretzschmar U. (2009). Potential alternative disinfection methods for organic fresh-cut industry for minimizing water consumption and environmental impact. LWT Food Sci. Technol. 42, 686–693. 10.1016/j.lwt.2008.08.001

[B64] Park E. Lee C. Bisesi M. Lee J. (2014). Efficiency of peracetic acid in inactivating bacteria, viruses, and spores in water determined with ATP bioluminescence, quantitative PCR, and culture-based methods. J. Water Health 12, 13–23. 10.2166/wh.2013.002 24642428

[B65] Pascual A. Llorca I. Canut A. (2007). Use of ozone in food industries for reducing the environmental impact of cleaning and disinfection activities. Trends Food Sci. Technol. 18, S29–S35. 10.1016/j.tifs.2006.10.006

[B66] Perry J. J. Yousef A. E. (2011). Decontamination of raw foods using ozone-based sanitization techniques. Annu. Rev. Food Sci. Technol. 2, 281–298. 10.1146/annurev-food-022510-133637 22129384

[B67] Rasimus S. Kolari M. Rita H. Hoornstra D. Salkinoja-Salonen M. (2011). Biofilm-forming bacteria with varying tolerance to peracetic acid from a paper machine. J. Ind. Microbiol. Biotechnol. 38, 1379–1390. 10.1007/s10295-010-0921-4 21161323

[B68] Rossi S. Antonelli M. Mezzanotte V. Nurizzo C. (2007). Peracetic acid disinfection: a feasible alternative to wastewater chlorination. Water Environ. Res. 79, 341–350. 10.2175/106143006X101953 17489268

[B69] Sapers G. M. (2001). Efficacy of washing and sanitizing methods for disinfection of fresh fruit and vegetable products. Food Technol. Biotechnol. 39, 305–311.

[B70] Schenk M. Raffellini S. Guerrero S. Blanco G. A. Maris Alzamora S. (2011). Inactivation of *Escherichia coli, Listeria innocua* and *Saccharomyces cerevisiae* by UV-C light: study of cell injury by flow cytometry. LWT Food Sci. Technol. 44, 191–198. 10.1016/j.lwt.2010.05.012

[B71] Schlüter O. Foerster J. Geyer M. Knorr D. Herppich W. (2009). Characterization of high-hydrostatic-pressure effects on fresh produce using chlorophyll fluorescence image analysis. Food Bioprocess Technol. 2, 291–299. 10.1007/s11947-008-0143-1

[B72] Schlüter O. Fröhling A. (2014). NON-THERMAL PROCESSING cold plasma for bioefficient food processing, in Encyclopedia of Food Microbiology, 2nd Edn. ed Tortorello C. A. B. L. (Oxford: Academic Press), 948–953.

[B73] Steudler S. Böhmer U. Weber J. Bley T. (2015). Biomass measurement by flow cytometry during solid-state fermentation of basidiomycetes. Cytometry A 87, 176–188. 10.1002/cyto.a.22592 25475642

[B74] Surowsky B. Fischer A. Schlueter O. Knorr D. (2013). Cold plasma effects on enzyme activity in a model food system. Innovative Food Sci. Emerg. Technol. 19, 146–152. 10.1016/j.ifset.2013.04.002

[B75] Surowsky B. Fröhling A. Gottschalk N. Schlüter O. Knorr D. (2014a). Impact of cold plasma on Citrobacter freundii in apple juice: inactivation kinetics and mechanisms. Int. J. Food Microbiol. 174, 63–71. 10.1016/j.ijfoodmicro.2013.12.031 24462703

[B76] Surowsky B. Schlüter O. Knorr D. (2014b). Interactions of non-thermal atmospheric pressure plasma with solid and liquid food systems: a review. Food Eng. Rev. 7, 82–108. 10.1007/s12393-014-9088-5

[B77] Tamburini S. Ballarini A. Ferrentino G. Moro A. Foladori P. Spilimbergo S.. (2013). Comparison of quantitative PCR and flow cytometry as cellular viability methods to study bacterial membrane permeabilization following supercritical CO_2_ treatment. Microbiology 159, 1056–1066. 10.1099/mic.0.063321-0 23579687

[B78] Thanomsub B. Anupunpisit V. Chanphetch S. Watcharachaipong T. Poonkhum R. Srisukonth C. (2002). Effects of ozone treatment on cell growth and ultrastructural changes in bacteria. J. Genet. Appl. Microbiol. 48, 193–199. 10.2323/jgam.48.193 12469318

[B79] Tiwari B. K. M. K. (2012). Ozone in fruit and vegetable processing, in Ozone in Food Processing, eds O'Donnell C. T. Tiwari B. K. Cullen P. J. Rice R. G. (West Sussex: Wiley-Blackwell), 55–80.

[B80] Vandekinderen I. Devlieghere F. De Meulenaer B. Ragaert P. Van Camp J. (2009). Optimization and evaluation of a decontamination step with peroxyacetic acid for fresh-cut produce. Food Microbiol. 26, 882–888. 10.1016/j.fm.2009.06.004 19835776

[B81] Van De Velde F. Gueemes D. R. Pirovani M. E. (2014). Optimisation of the peracetic acid washing disinfection of fresh-cut strawberries based on microbial load reduction and bioactive compounds retention. Int. J. Food Sci. Technol. 49, 634–640. 10.1111/ijfs.12346

[B82] Vleugels M. Shama G. Deng X. T. Greenacre E. Brocklehurst T. Kong M. G. (2005). Atmospheric plasma inactivation of biofilm-forming bacteria for food safety control. IEEE Trans. Plasma Sci. 33, 824–828. 10.1109/TPS.2005.844524

[B83] Wang H. Feng H. Lao Y. (2006). Dual-phasic inactivation of *Escherichia coli* O157:H7 with peroxyacetic acid, acidic acid, acidic electrolyzed water and chlorine on cantaloupes and fresh-cut apples. J. Food Saf. 26, 335–347. 10.1111/j.1745-4565.2006.00053.x

[B84] Zhang Y. Q. Wu Q. P. Zhang J. M. Yang X. H. (2011). Effects of ozone on membrane permeability and ultrastructure in *Pseudomonas aeruginosa*. J. Appl. Microbiol. 111, 1006–1015. 10.1111/j.1365-2672.2011.05113.x 21790913

